# A reproducible systematic map of research on the illusory truth effect

**DOI:** 10.3758/s13423-021-01995-w

**Published:** 2021-10-27

**Authors:** Emma L. Henderson, Samuel J. Westwood, Daniel J. Simons

**Affiliations:** 1 Faculty of Business and Social Sciences, Kingston University, Kingston Hill Campus, Kingston Hill, Kingston upon Thames, KT2 7LB UK; 2 Department of Psychology, Institute of Human Sciences, Millennium City Building, University of Wolverhampton, Wolverhampton, WV1 1LY UK; 3 Department of Child & Adolescent Psychiatry, Institute of Psychiatry, Psychology and Neuroscience, King’s College London, London, SE5 8AB UK; 4 Department of Psychology, University of Illinois, 603 E. Daniel Street, Champaign, IL 61820 USA

**Keywords:** Illusory truth effect, Repetition, Truth judgement, Systematic map, Transparency, Registered report

## Abstract

People believe information more if they have encountered it before, a finding known as the *illusory truth effect*. But what is the evidence for the generality and pervasiveness of the illusory truth effect? Our preregistered systematic map describes the existing knowledge base and objectively assesses the quality, completeness and interpretability of the evidence provided by empirical studies in the literature. A systematic search of 16 bibliographic and grey literature databases identified 93 reports with a total of 181 eligible studies. All studies were conducted at Western universities, and most used convenience samples. Most studies used verbatim repetition of trivia statements in a single testing session with a minimal delay between exposure and test. The exposure tasks, filler tasks and truth measures varied substantially across studies, with no standardisation of materials or procedures. Many reports lacked transparency, both in terms of open science practices and reporting of descriptive statistics and exclusions. Systematic mapping resulted in a searchable database of illusory truth effect studies (https://osf.io/37xma/). Key limitations of the current literature include the need for greater diversity of materials as stimuli (e.g., political or health contents), more participants from non-Western countries, studies examining effects of multiple repetitions and longer intersession intervals, and closer examination of the dependency of effects on the choice of exposure task and truth measure. These gaps could be investigated using carefully designed multi-lab studies. With a lack of external replications, preregistrations, data and code, verifying replicability and robustness is only possible for a small number of studies.

**Supplementary Information:**

The online version contains supplementary material available at 10.3758/s13423-021-01995-w.

## Introduction

“Sixty-two thousand four hundred repetitions make one truth.”

-- Aldous Huxley (1932), Brave New World (p. 46)

With this satirical statement, Huxley highlights the power of repetition to manipulate belief. Repetition can increase subjective truth judgements, a phenomenon known as the “illusory truth effect.” The effect of repetition on belief occurs for both true and false statements (Brown & Nix, 1996), for both plausible and implausible ones (Fazio, Rand, & Pennycook, 2019), and for both known and unknown information (Fazio, Brashier, Payne, & Marsh, 2015). It appears with only minutes between repetitions (Unkelbach & Greifeneder, 2018), and with delays of weeks (Gigerenzer, 1984) and even months (Brown & Nix, 1996). Although most studies use sets of trivia statements, it apparently works for consumer testimonials (Roggeveen & Johar, 2002), statements of opinion (Arkes, Hackett, & Boehm, 1989), and false news stories (Polage, 2012). If the illusory truth effect truly generalizes beyond the lab, it might help explain the use of repetition to override facts in propaganda campaigns (Lewandowsky, Stritzke, Oberauer, & Morales, 2005; Paul & Matthew, 2016; Pennycook, Cannon, & Rand, 2018). By the same token, it seems that information can enter the public lexicon through repetition rather than accuracy. Familiarity can apparently trump rationality. But what is the evidence for the generality and pervasiveness of the illusory truth effect?

Over the past few years, awareness of the illusory truth effect has grown, with articles in *Vox*, *The Atlantic*, and *Wired* (Dreyfuss, 2017; Paschal, 2018; Resnick, 2017) linking it to “fake news,” “truthiness,” and President Trump’s communication style. Yet the only meta-analytic review of this literature appeared in 2010 (Dechêne, Stahl, Hansen, & Wänke, 2010). It combined the results of 51 studies conducted before 2008, and it estimated a medium effect size: (*d* = .39; 95% CI: [0.30, 0.49]) within-items, *d* = .50; 95% CI: [0.43, 0.57]) between-items, random effects model). The meta-analysis is somewhat dated, both because new studies have been published and because it was completed prior to recent advances in techniques used to address publication bias.

Publication bias is prevalent in psychology. Approximately 95%^1^ of published articles contain statistically significant confirmation of the stated hypothesis (Fanelli, 2010; Sterling, Rosenbaum, & Weinkam, 1995). Synthesizing the results from a biased pool of research, dominated by significant, “positive” findings, threatens the validity and interpretation of results, and in meta-analyses it also makes the overestimation of effect sizes likely (Renkewitz & Keiner, 2019). Although Dechêne et al. (2010) note that a funnel plot for the analyzed studies appeared symmetrical, their article did not include the funnel plot or any formal analyses of it, and it is possible that other bias correction approaches would estimate a smaller effect.

We originally preregistered a plan to conduct an updated meta-analysis of the illusory truth effect (https://osf.io/j6fmr/). As part of the pilot testing in that plan, intended as a first stage to help develop an appropriate coding scheme, the first and third authors, along with an additional coder, each independently coded a random selection of papers from those included in the 2010 meta-analysis. It quickly became apparent that these papers did not report sufficient information to estimate the observed effect size for the illusory truth effect without making strong, questionable assumptions. For example, the selected papers did not consistently report inferential statistics for the main effect of repetition (the illusory truth effect), included no variance estimates, and/or obscured the effect of interest by combining groups into a more complex analysis.^2^ Dechêne et al. (2010) encountered the same issues of under-reporting and described the assumptions they made in order to address them in their meta-analysis:

“Twenty-one studies provided standard deviations for the reported means; seven studies reported a range of standard deviations. In the latter case, we computed the pooled standard deviations from the range. Where no standard deviations were provided [*23 studies, 45% of the sample of studies*], we chose to impute the pooled standard deviation from an overall estimate that was obtained from those studies in which standard deviations were reported or could be extracted” (Dechêne et al., 2010, p.243; text within brackets added).

The extent of the issue was unclear, though, because the paper did not specify the number of effects that required imputed variance estimates.

In our view, these assumptions cloud conclusions about the overall strength and consistency of the evidence for the illusory truth effect. Imputing estimates of variance when computing standardized effect sizes is suboptimal for at least two reasons: First, it is possible that the subset of studies that do report information about variance differ systematically from those that do not. For example, the studies that report variance might have been more rigorous and precise in their measurement practices, leading to smaller variance estimates and larger standardized effects. If so, using their variance estimates for other studies would yield inflated overall effect estimates. Second, studies with different designs may not have similar variance estimates. For example, variance estimates will differ with the number and type of experimental items and the breadth of the scale used to measure truth ratings (e.g., dichotomous, 1–6, or a continuous response slider). Unfortunately, Dechêne et al. (2010) could not provide us with the coded data that were used to produce their 2010 meta-analytic estimates, and many of the studies included are old (~30 years), making the original data unavailable. Based on our coding attempt, the lack of available data, and the need to make overly strong assumptions in order to estimate effects for many of the published papers, we concluded that a valid meta-analysis is not possible for the entirety of this literature.

Given these challenges, we chose instead to create a systematic map; a method of evidence synthesis designed to assess the nature of a literature base (Haddaway et al., 2019). The primary objective of a systematic map is to locate and catalogue the breadth of evidence on a particular topic using predetermined, transparent, and reproducible methods (Haddaway, 2018). Systematic maps can thereby answer questions such as: how many studies have been conducted? Which methods were used? What is the mean sample size used? The output from a systematic map is an accessible, searchable database(s) that can then be used by the research community. Specifically, the database can be used to highlight knowledge clusters, knowledge gaps, areas with limited or weak evidence (Corker, 2018), or investigations of particular combinations of variables. Systematic maps differ from systematic reviews or meta-analyses in that they do not attempt to answer specific questions about the effectiveness of an intervention, the truth or falsity of a hypothesis, or to estimate effect sizes. Rather, systematic maps have an open framing that allows a wider range of evidence to be summarized in the database (James, Randall, & Haddaway, 2016). For a comparison of systematic maps and systematic reviews, see James et al. (2016) Table 1. Systematic mapping is particularly useful for domains with a wide range of experimental manipulations (e.g., different delays, different types of items) tested in a wide range of contexts and with different measures (James et al., 2016). We created two inter-related databases: an abstract-level database that includes relevant articles where the full text could not be obtained, and an extensively coded full-text database.

**Table 1:** List of bibliographic and grey literature databases/platforms searched along with the search fields

	Type	Database	Field	Comments
1	Bibliographic	Business Source Premier (EBSCOHost)	“Abstract or author-supplied abstract”	Using “Advanced Search”
2	Bibliographic	EconLit (EBSCOHost)	“Abstract”	Using “Advanced Search”
3	Bibliographic	ERIC (EBSCOHost)	“Abstract”	Using “Advanced Search”
4	Bibliographic + Grey	Google Scholar	“The phrase”	Accessed via Publish or Perish
5	Bibliographic	PsycINFO (Ovid)	“Abstracts”	Using the “Advanced Search”
6	Bibliographic	PubMed (NCBI)	“Title/Abstract”	Using “Advanced Search Builder”
7	Bibliographic	Scopus (Elsevier)	“Article title, Abstract, Keywords”	Using “Advanced Search”
8	Bibliographic	Web of Science	“Topic”	Using “Basic Search”
9	Theses and conference papers	OpenGrey		
10	Preprints	PsyArXiv (OSF Preprints)		
11	Replications	Curate Science		
12	Replications	PsychFileDrawer		
13	Theses	DART-Europe		
14	Theses	EthOS (British Library)		
15	Theses	ProQuest Dissertation & Theses Global (ProQuest)		
16	Theses	Thesis Commons (OSF Preprints)		

*Note*. The interface or platform through which the database was searched is in parentheses. The Web of Science platform was used to search the following collections: Web of Science Core Collection, BIOSIS Citation Index, BIOSIS Previews (until 2008 only), KCI-Korean Journal Database, MEDLINE, Russian Science Citation Index, SciELO Citation Index

In addition to producing a traditional systematic map, we assessed the transparency and reproducibility of the empirical studies identified by the map. Transparency and reproducibility are the cornerstones of the scientific method and knowledge generation. Recent concerns about poor transparency and low reproducibility have catalyzed open practices and reforms designed to enable more transparent science (Munafò et al., 2017; Nosek et al., 2015). Meta-research has begun to evaluate adoption of reforms across broad areas, for example in research in social sciences (Hardwicke et al., 2020). Here we assess the statistical, methodological, and reporting practices that may impact the robustness of conclusions that can be drawn from the entirety of a single research area. We coded a number of indicators of transparency and reproducibility. For example, the availability of raw data, the provision of which allows computational reproducibility. We also coded whether the main effect of repetition was reported as observed/significant/marginally significant/non-significant by the authors as a proxy measure for publication bias; a published literature without bias towards significant results should be characterized by a mix of both significant and non-significant results. A full list of the variables coded is detailed in Table 2.

**Table 2:** Summary of study characteristics extracted and coded

	Variable	Details/examples	Variable described in text
	General information (article level)		
1-5	Bibliographic information	APA citation, Author, Year, Title, Journal	Y (partially)
6	Google Scholar link		N
7	Document type	Journal article, PhD thesis, MSc dissertation, conference paper, poster, book chapter, unpublished article, unpublished data, unpublished preprint	Y
8	Publication status	Was the study published in a peer-reviewed journal?	Y
9-10	Citation count	ELH coded the citation count on a single day using Web of Science and Google Scholar	N
11	Source	How was the study or these data first located?	N
12	Subject area	What is the broad subject area?	Y
13	Evidence synthesis	Has the study been included in a previous evidence synthesis?	Y
14	Retraction	Has the paper been retracted? (http://retractionwatch.com)	Y
15	Language	In which language is the article written?	Y
16	Number/name of coders	Report who coded the study	N
17	Full text^b^	Is the full text of the article available?	N
18	Study country	Which country is the corresponding author based in according to their affiliation?	Y
19	Number of studies	How many studies does the article report?	N
20	Number of illusory truth effect studies	How many of the studies relate to the illusory truth effect?	N
	Open research practices (article level)		
21	Replication	Does the article claim to report a replication study?	Y
22	Preregistration	Does the article report a study (or some aspect of a study) that was preregistered?	Y
23	Preregistration located	Where does the article indicate the study was preregistered?	N
24	Open data	Does the article state whether or not data are openly available?	Y
25	Raw data	Can you access, download, and open the raw data files?	Y
26	Open analysis scripts	Does the article state whether or not analysis scripts are available?	Y
27	Open materials	Does the article state whether or not materials are available?	Y
28	OSF	Were any additional data files or materials shared on the OSF?	Y
29	Article access	Is the article available open access (using https://openaccessbutton.org/)?	Y
30	statcheck	Can statcheck (http://statcheck.io/) read the PDF?	Y
31	statcheck checked	Report number of statistics checked by statcheck	N
32	statcheck issues^c^	Report number of issues highlighted by statcheck	supplement
33	Links	Links to preregistrations, open data, code, or materials	N
	Study design (study level)		
34	Experimental aim^d^	Describe the main aim/purpose of the study	N
35	Goal vary ITE	Did the abstract state that the primary goal of the study was to vary the magnitude of the overall ITE effect by varying some factor (moderation/mediation)?	Y
36	Results vary ITE	Did the abstract report finding evidence that the magnitude of the overall ITE varied as a function of a manipulated variable?	Y
37	Overall test ITE	In the abstract, do the authors describe the outcome of the overall test of what they define as the illusory truth effect?	Y
38	Sample size tested^d^	Number of participants tested	Y
39	Sample population	Which population made up the study sample?	Y
40	Study design	Was repetition manipulated within- or between-subjects?	N
41	Design^d^	Describe the overall factorial design of the study	N
42	Within-subjects factors^d^	Describe the within-subjects factors and groups	N
43	Between-subjects factors^d^	Describe the between-subjects factors and groups	N
44	Stimuli type	Type of experimental stimuli	Y
45	Study setting	In which setting was the study conducted (e.g., lab, online)?	Y
	Exposure session(s) (study level)		
46	Stimuli presentation exposure	How were the stimuli presented during exposure phase (e.g., auditory, visual)?	N
47	Repetitions manipulated exposure	Were the number of repetitions manipulated during exposure phase?	Y
48	Number of repetitions exposure^d^	Number of times participants are exposed to statements during exposure phase(s)	Y
49	Tasks exposure^d^	List all tasks completed with the critical items during exposure phase(s)	Y
	Retention interval (study level)		
50	Retention interval^d^	Time between exposure and (each) test phase(s)	Y
51	Filler task^d^	List any task(s) completed during retention interval	Y
	Test session(s) (study level)		
52	Repetition type	Were the statements repeated verbatim or gist?	Y
53	Stimuli presentation test	How were the stimuli presented during test phase (e.g., auditory, visual)?	N
54	Statement mix	At test were all statements repeated, or a mix of old and new?	N
55	Number of test sessions^d^	Number of test sessions (excluding exposure phase(s))	Y
56	Number of repetitions test^d^	Total number of exposures across all test phases	Y
57	Truth measure	Type of truth measure used as the dependent measure	Y
58	Prior knowledge	Does the study test whether participants already knew the answers to test items prior to the study?	Y
	Results (study level)		
59	Overall test reported	Do the authors report a single overall test of what they define as the illusory truth effect?	Y
60	Measurement design	How was the overall illusory truth effect measured (i.e., between/within-items)?	N
61	Test statistic^d,e^	Report the test statistic for the overall effect of illusory truth	N
62	Degrees of freedom^d,e^	Report degrees of freedom for main effect of illusory truth	N
63	Reported p-value^d,e^	Report p-value for main effect of illusory truth	N
64	Calculated p-value^d,e^	Report calculated p-value from statcheck	N
65	Direction of test^e^	Report whether the statistical test was specified as one-sided or two-sided	N
66	Effect size^d,e^	Report the type and value of the effect size for main effect of illusory truth	Y
67	Confidence interval^d,e^	Report the confidence/credible interval for the effect size	N
68	Overall test significant^e^	Do the authors report in their prose in the results section that the overall test of illusory truth effect was observed/statistically significant/marginally significant/non-significant?	Y
	Sample size and transparent data reporting (study level)		
69	Sample size justification	Does the study report a justification for the choice of sample size?	Y
70	Statistical sampling plan	Does the study report a formal power analysis or Bayesian sampling plan?	Y
71	Exclusions reported^e^	Does the study report where participants, or data within participants, were excluded from analysis?	Y
72	Exclusions number reported^d,e^	How many participants does the study report as being excluded?	N
73	Means^e,f^	Does the study report means for critical conditions?	Y
74	Measures of variance^e,f^	Does the study report the variance (or SDs) for the means of critical conditions?	Y

*Note. Y* means that the variable is reported in the text of this paper. *N* means that the variable can be found in the systematic map database

^b^ Articles in the abstract-level database were not coded beyond this variable

^c^ We reported the statcheck results without further evaluation. Where statcheck was able to read the PDF, summary reports are available on the OSF

^d^ Indicates variables coded using free-text rather than dropdown options

^e^ Indicates variables that were not coded if the study did not report a focused test of new vs. repeated statements (i.e., a main effect for repetition)

^f^ Where inferential statistics were reported

For changes between the Stage 1 approved coding scheme and the final coding scheme please see https://osf.io/a9mfq/

### Research aims

Illusory truth effect research typically follows a standard paradigm: Participants first read or hear a number of statements, normally trivia statements, during an exposure phase. At test, participants judge the truth of a set of statements generally comprised of half old statements (repeated from the exposure phase) and half new statements (previously unseen). However, these studies can vary in a number of ways. For example, they might measure the truth effect as the difference in truth ratings from exposure to test phase (within-items), or as the difference in truth ratings between new and repeated statements at test (between-items). At exposure stage, they might ask participants to simply read the statements (Unkelbach & Rom, 2017) or rate them for familiarity (Garcia-Marques, Silva, & Mello, 2017). They might test clinical or non-clinical populations, use one or multiple repetitions, or introduce no delay or a long delay between repetitions.

The primary aim of this research was to systematically identify and map published and unpublished research examining the relationship between repetition of statements and subjective truth ratings with the following objectives:

1. Describe the current nature and extent of the literature on the topic.
2. Assess the transparency and reproducibility of the literature (using the objective measures described below).
3. Collate and highlight any well-represented subtopics (e.g., studies that use trivia statements as stimuli) that might benefit from more detailed secondary research (knowledge clusters).
4. Identify knowledge gaps in the evidence base (i.e., areas that have not been frequently studied).
5. Provide direction for novel research or single/multi-lab replication studies in which outstanding questions can be empirically tested.
6. Produce a systematic map that is transparent, reproducible, and open so that it may be used and updated by others (Lakens, Hilgard, & Staaks, 2016).^3^

## Method

### Conformance with reporting and quality standards

In preparing the systematic map protocol, we adhered to the Preferred Reporting Items for Systematic reviews and Meta-Analyses Protocols (PRISMA-P; Moher et al., 2015; Shamseer et al., 2015) and the RepOrting standards for Systematic Evidence Syntheses (ROSES; Haddaway, Macura, Whaley, & Pullin, 2018b).^4^ The completed ROSES form for systematic review protocols is available at https://osf.io/ux2vz/. In our reporting of systematic searches, we followed the PRISMA-S extension for the reporting of systematic review searches (Rethlefsen et al., 2021). The meta-data are open with fully reproducible analyses, coded data files, analysis scripts, and supplementary materials available at https://osf.io/dm9yx/.

### Search term identification and selection

All steps in this search term identification and selection section were completed prior to submitting the Stage 1 Registered Report. We defined our search terms with the assistance of the R package litsearchr (Grames, Stillman, Tingley, & Elphick, 2019b). Litsearchr reduces bias in keyword selection by partially automating the selection process (Grames, Stillman, Tingley, & Elphick, 2019a). Litsearchr uses a keyword extraction algorithm to locate potential keywords from a sample of papers and combines them with author and database tagged keywords to create a list of potential keywords. Important keywords are identified from their predominance in a keyword co-occurrence network.

#### Scoping search

First, a scoping search was conducted using Scopus and Web of Science and the below search string. Searches were conducted on 14 June 2019 with no date restrictions. The number of hits were as follows: Scopus (156), Web of Science (63).

- (“illusory truth” OR “illusory truth effect” OR “illusions of truth” OR “reiteration effect” OR “repetition induced truth effect” OR “repetition based truth effect” OR “truth effect” OR "truth judgment")

#### Litsearchr

The results of the scoping search were imported into R. N-grams that occurred at least three times in the dataset and in a minimum of three studies were extracted and coded as relevant/irrelevant to the search. The same process was followed for similar terms. The litsearchr code and resulting files can be found at https://osf.io/hdtgb/. We incorporated the additional terms identified by the litseachr package, along with relevant unigrams into the search string.

#### Testing the comprehensiveness of the search

To estimate the comprehensiveness of the search, we compiled a set of 20 papers of known relevance to the review to serve as a benchmark list (see Appendix A). We conducted a scoping search, using Web of Science and Scopus, to ensure that all 20 papers were indexed and captured by the search terms. For any papers that were not initially found, we identified the reasons why they were missed, adjusted the search string accordingly, and checked that the string now captured those papers. The search string below is shown as formatted for Web of Science (exact search strings by database are documented in Appendix B):

- (((“illusory truth” OR “illusion* of truth” OR “induced truth effect” OR “reiteration effect” OR “tainted truth effect” OR “repetition based truth effect” OR “repetition induced increases” OR repeat OR repeated OR repeating OR repetition OR “prior exposure”) AND (true* OR truth OR “truth effect*” OR belief) AND (statement* OR items OR stimulus OR stimuli OR claim* OR judgment* OR judgement* OR rating* OR “subjective truth” OR “truth value” OR “judged validity” OR “validity ratings” OR “processing fluency” OR “fluency effect*” OR “perceptual fluency”)))

### Search strategy

A summary of the workflow for our search strategy can found be at https://osf.io/f9462/. Using the predefined search string we carried out an extensive literature search that aimed to minimize the effect of publication bias on our map. Considerable effort was devoted to searching for both published and unpublished studies, as well as replications. We consulted an academic librarian for advice on the details of our scoping search terms and search strategy. The electronic searches were conducted by the first author on 4 and 6 February 2020 without any limits or restrictions. Any articles published after that date were not included.^5^ We preregistered that if the review took more than 2 years to complete, we would update the searches. All searches and outcomes were recorded in a Search Record Appendix (https://osf.io/xsnhm/). Table 1 outlines further details of the fields used for each search.

#### Electronic bibliographic database searches

First, a comprehensive computerized search of illusory truth studies was performed using the above search string in eight bibliographic databases/platforms. This selection of databases includes all seven of those used in the Dechêne et al. (2010) meta-analysis (see Table 1 and Appendix B).

#### Grey literature searches

Furthermore, we included grey literature by searching for items such as doctoral theses, conference papers, preprints, and replication attempts in eight databases (see Table 1). Google Scholar has been identified as effective in retrieving grey literature (Haddaway, Collins, Coughlin, & Kirk, 2015) and was used to supplement the other search methods. To increase reproducibility we used Publish or Perish (Harzing, 2007) to carry out searches and export the results. Because Google Scholar allows only basic Boolean operators in search strings, the search string was reduced to the key components detailed in Appendix B. Search strings for all other grey literature sources are also documented in Appendix B.

#### Researcher-to-researcher channels

Upon completing the electronic searches, we issued calls for unpublished studies through the Listservs of the Society for Personality and Social Psychology (SPSP), the Society for Judgment and Decision Making (JDM), Psychonomic Society, Cognitive Science Society (CSS), European Association of Social Psychology (EASP), and the Society for Consumer Psychology (SCP).^6^ We issued one call per society. If the calls led to direct correspondence with a researcher, we asked them to send us any (other) unpublished studies directly. Simultaneously, we posted notices on Twitter (twice each week for 3 weeks) and included a link to a public Google document to allow researchers to suggest additional citations.

Finally, once eligible papers from the database and grey literature searches had been identified through full-text screening, we contacted corresponding authors for any preprints, or unpublished studies/papers that they were aware of and any published studies we might have missed. We used the email address provided in the paper. If the email was returned undelivered, we searched online for a current email address. If none could be found, we tried to reach the other authors. If authors did not respond to the initial email, 2 weeks later a second email offered the chance to provide unpublished studies anonymously using a file transfer service. We did not send further request emails. A record of the correspondence (who was contacted, on which date, the general nature of the response) was retained. We kept this record private but report the response rates. The wording of emails and the Listserv message can be found at https://osf.io/52c4q/.

After initiating an email correspondence with a researcher, either as a corresponding author who might have unpublished studies or as a response to a Listserv contact, we allowed 10 weeks (from the date of the first email) to receive studies from them. Even where relevant studies were received after 10 weeks, we were able to include them in the map.

As a result of our calls, three authors contacted us via Twitter and four authors responded to Listserv messages. Of the remaining 46 first authors, we were able to contact 32, and 23 responded. From this correspondence, eight authors offered potentially relevant papers, resulting in 21 additional papers that eventually were included in the map.

#### Manual searches

Once relevant meta-analyses and review articles had been identified during title/abstract screening, their reference lists were manually screened for supplementary papers (i.e., *backward search*). Upon completion of full-text screening, we also manually reviewed the bibliographies of the eligible papers for any additional studies that had not been captured by the database searches. Additional papers identified via manual searches were screened at the full-text level.

#### Reproducibility of unpublished studies

Unpublished studies pose a threat in terms of reproducibility and the cumulative updating of a systematic map. For any unpublished studies we received, we asked the author’s permission to share the unpublished report/data/summary. In all cases, authors agreed to share either the whole report or a summary.

### Inclusion criteria

Since systematic maps are designed to give an overview of the topic area, they adopt broad inclusion criteria. We included articles that adhered to all of the following criteria:

1. *Population*: human populations of any age, including those from clinical groups
2. *Intervention*: verbatim or gist repetition of multiple statements (e.g., trivia, political, marketing) presented visually or aurally
3. *Comparator*: within-subjects (repeated vs. non-repeated statements) or between-subjects (non-repetition control vs. repetition group)
4. *Outcome*: numerical (Likert-type scale, slider, or similar) or binary (true/false) measures of subjective truth judgements, either comparing truth ratings made before and after repetition (within-items), truth ratings for new versus repeated items (between-items), or non-repetition control versus repetition group (between-subjects)
5. *Study type*: empirical quantitative studies
6. *Time frame*: no constraints

### Exclusion criteria

At the title and abstract screening stage, excluded papers were simply marked as “no.” At full-text screening stage a list of excluded articles is reported along with a specific reason from the list below. The list illustrates the sequence in which exclusion criteria were applied. Therefore, if an article could have been excluded for multiple reasons, we required that only one reason be given (i.e., the first criterion at which it fails). We excluded studies for the following predefined reasons:

1. *Population*: non-human population
2. *Study type*: review paper^7^
3. *Study type*: no quantitative data
4. *Intervention*: the study did not use repetition as a manipulation to increase subjective truth judgements
5. *Outcome*: the study did not measure subjective truth judgements
6. *Comparator*: the study did not compare ratings for repeated versus non-repeated statements, or ratings from a non-repetition control group with those from a repetition group
7. *Other*: entirely superseded by a later paper. Multiple reports of the same study were collated into a superset and coded as one unit. Papers were only excluded where it was clear that the earlier version contained no additional information. Specifically, this refers to cases in which a study described in a preprint, dissertation, Stage 1 Registered Report, or conference abstract/presentation was fully reported in a later paper. In cases of partial overlap (e.g., a paper that reports only three of the four studies included in a dissertation), the reports were connected in the database to ensure that all studies were coded
8. *Other*: any dataset that was not accompanied by descriptive meta-data detailing the methods used to test the illusory truth effect (e.g., unpublished data received via contact with authors) were excluded from the full-text database because the information needed to code the study was missing. However, it was included in the abstract-level database
9. *Other*: the paper was written in a language other than English or French and a translator could not be recruited

In addition to the above preregistered exclusion criteria, if an abstract was incomplete during the title/abstract screening stage and we subsequently retrieved the full abstract, we first reviewed that complete abstract during full-text screening, and if it was excluded, we coded it using the additional criterion, “screened abstract – not relevant.”

### Study screening procedure

The publications returned from the electronic searches were imported into Zotero. Duplicate references were identified and removed using Zotero’s “duplicate items” feature based on title, DOI, and ISBN fields. In cases of dual publication (e.g., a conference paper or PhD thesis later published in a peer-reviewed journal), we extracted the superset of studies in case each had content that the other did not. For the purpose of maintaining records, we kept a comprehensive list of all references before duplicates were removed.

The deduplicated records were then imported into *Covidence*, Cochrane’s online systematic review tool that facilitates collaborative screening. We followed a two-stage screening process: Initially two coders independently screened the titles and then the abstracts using Covidence and the predefined inclusion and exclusion criteria. Studies were coded as (1) yes, (2) no, or (3) maybe. A paper was coded “maybe” if insufficient information was available to enable an eligibility decision or if there was doubt about the presence of an inclusion criterion. In this case, the paper was retained and a decision made at the full-text stage. Screening decisions were compared using Cohen’s Kappa. Scores of 0.64 (ELH and DJS) and 0.60 (ELH and FVT) were obtained, indicating substantial agreement. Covidence highlights any discrepancies in a section called “resolve conflicts.” Any conflicts were reviewed by the first author and resolved by discussion with the relevant coder.

We then retrieved the full text of each paper. Each article was downloaded in PDF format from whatever source was available (e.g., journal website, interlibrary loan, author website, email to the corresponding author, British Library). If the full text was unavailable, the article was still coded, but in the abstract-level database only. Once full texts had been retrieved, coders independently used Covidence to apply the inclusion and exclusion criteria based on a brief evaluation of the full text. The Cohen’s Kappas for full-text screening were 0.94 (ELH and DJS) and 0.61 (ELH and FVT). Any disagreements about either the inclusion/exclusion decision or the reason for exclusion were discussed between the two coders, and any remaining disagreements were adjudicated by the remaining coder. A record of full-text evaluations is available at https://osf.io/xsnhm/. Once full-text eligibility screening was complete, we carried out the additional manual searches of bibliographies and contacted corresponding authors, as detailed in the search strategy section.

**Fig. 1:**
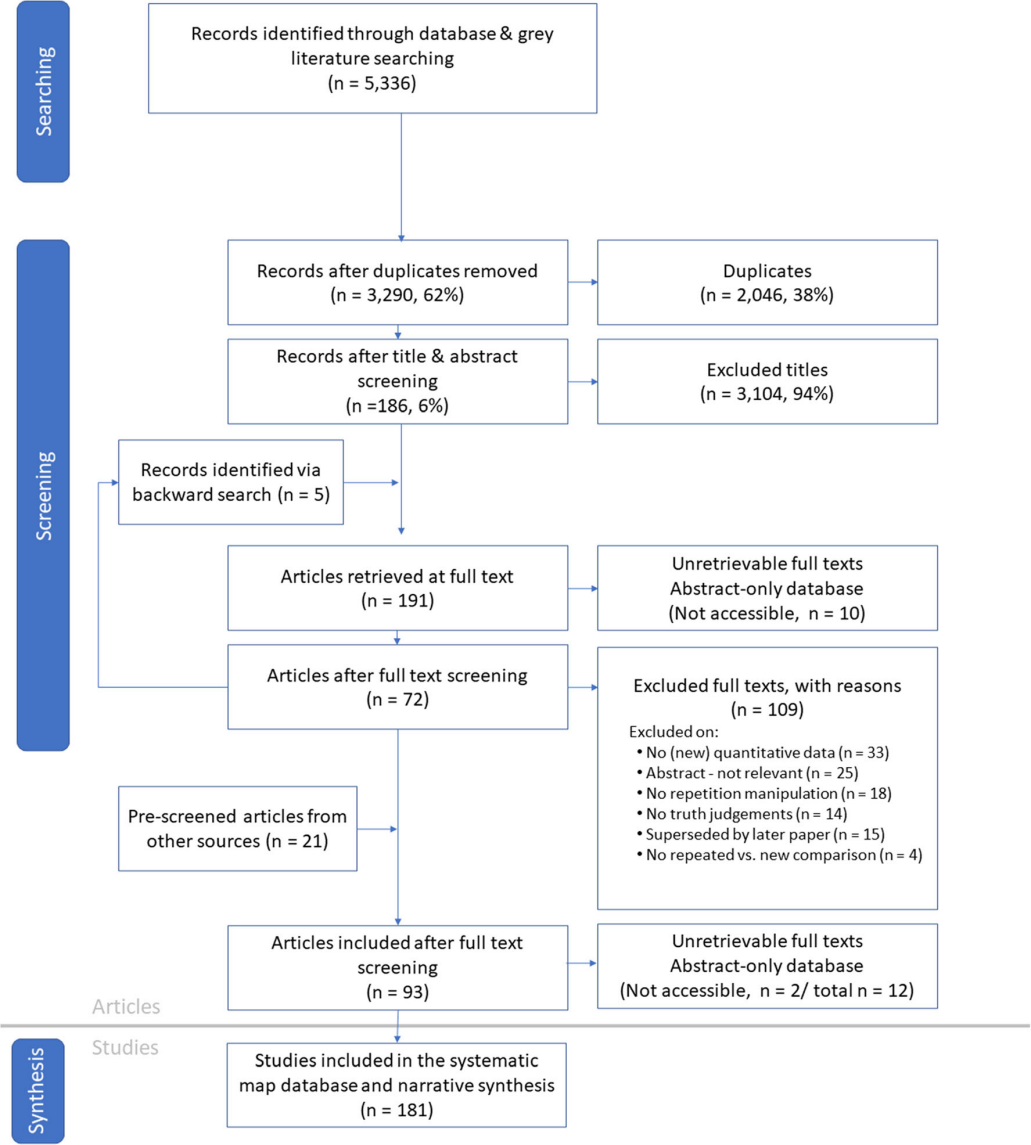
ROSES (RepOrting standards for Systematic Evidence Syntheses) flow diagram for systematic maps (version 1.0)

We used the ROSES flow diagram for systematic maps (Haddaway, Macura, Whaley, & Pullin, 2018a) to report the flow of articles through all stages of the process from searching to synthesis for the systematic map (Fig. 1).

### Map coding and inter-rater reliability

Two inter-related databases were created in Excel files. The abstract-level database includes articles that appear to be relevant but where the full text could not be obtained. These articles were coded for bibliographic information only. To produce the full-text database, we extracted data from full-text articles using the coding scheme outlined below (see Table 2). If multiple studies were reported within one article, each study was coded on a separate line. Studies included only in appendices or described as pilot data were coded and flagged when enough information was provided to do so.

The coding scheme was split into article-level (Table 2, codes 1–33) and study-level codes (Table 2, codes 34–74). Data entered at the article-level included information such as citation count, study language, and the reporting of open research practices. None of the article-level codes required a judgement call, and the first author single-coded them.

At study level, initially we independently double-coded 30 papers. Each author coded ten papers with each other coder, resulting in 20 papers coded by each author. Papers were randomly chosen by executing the below commands in R:

- set.seed(123)
- sample(112, size = 30, replace=FALSE)

The first ten of these papers were coded by DJS and ELH, the next ten by DJS and SJW,^8^ and the remaining ten by ELH and SJW. After the coding was complete, we identified all disagreements and jointly evaluated whether they resulted from ambiguities in the coding instructions or from coding errors. For any cases of ambiguity, we reviewed the coding *instructions* and adjusted them. Each pair of authors then coded those previously ambiguous variables using the adjusted instructions for an additional set of five randomly selected papers. Where disagreements on interpretation remained, we repeated this process and coded a new set of five papers. This process iterated until the authors reached 100% agreement that the coding instructions were unambiguous and that they led to consistent coding (i.e., codes for dropdown menus exactly matched and codes for free text variables other than “notes” columns semantically matched). The changes to the coding instructions during this iterative process were documented and are reported at https://osf.io/a9mfq/.

Once 100% agreement was reached on the final set of coding instructions, the second author coded 20 additional papers, and all of the remaining papers were coded by the first author. By reducing ambiguity, we aimed to make our coding scheme as reproducible as possible. Even so, no coding scheme is perfect for every paper, and cases that the coder felt were ambiguous were discussed with either of the other authors, depending on availability at the time (such cases are documented in the coding file).

Coders highlighted the text for each coded variable in the article PDF files.^9^ Highlighted electronic copies of the extracted articles (PDFs) have been made as publicly available as possible given copyright restrictions.^10^ Following data extraction (at Stage 2), we approached the publishers (and authors for unpublished work) of all extracted articles to seek permission to archive the highlighted PDFs publicly, on the Open Science Framework (OSF). Two publishers (Instituto Superior de Psicologia Aplicada and University of Illinois Press) approved the request, but the majority of the publishers declined (APA, Elsevier, MIT Press, Oxford University Press, Springer, Taylor & Francis, and Wiley). We received no response from three other publishers (Chicago Press, Guildford Press, Sage). We therefore placed the annotated PDFs in a password-protected zip archive that is stored at https://osf.io/3hzmf/. The password will be provided upon request.

Table 2 summarizes the study characteristics we extracted and coded. We did not contact authors for additional information. The planned coding scheme is detailed in the “codingScheme_stage1RR_2ndrevision” Excel file https://osf.io/h2e5g/. We piloted the coding scheme by coding randomly selected papers from the reference section of Dechêne et al. (2010) and iteratively adapting the coding scheme. The pilot was preregistered on the OSF (https://osf.io/d7tb5). Additionally, the coding scheme was updated based on reviewer input during review of the Stage 1 Registered Report submission. The final coding scheme is available at https://osf.io/a9mfq/.

Where possible, our predefined coding scheme used dropdown menus to constrain data entry. For variables that we expected to be idiosyncratic (e.g., retention interval between exposure and test sessions) we entered data as free text. The free text variables are highlighted in Table 2. Once coding was complete, we merged any codes that used different terms for the same content to ensure consistent labelling. We then reviewed the free-text coding to determine whether meaningful clusters could be grouped for simplification. Such groupings are reported in the *Results* section, and in files at https://osf.io/ebnm5/.

The following broad categories of data were extracted for coding at either article or study level:

1. Bibliographic information
2. Methodological information about the study design, stimuli, and subjects
3. Information about the number of repetitions and delay between exposure and test phases
4. Study outcome
5. Level of adherence to transparent data reporting and open science practices.

## Results

The analysis script was written in R Markdown (Allaire et al., 2020). Analyses used R version 4.0.3 (R Core Team, 2019) with the packages plyr 1.8.6 (Wickham, 2011) for recoding variables, and tidyverse 1.3.0 (Wickham et al., 2017) for data wrangling and visualisation. See the “Data & Analysis” component on the OSF.

### Evidence identification, retrieval, and screening

The ROSES diagram (Fig. 1) summarizes the steps involved in this systematic map and the number of articles added or excluded at each stage. The 5,336 potentially relevant results from bibliographic and grey literature searches (4 and 6 February 2020) resulted in 3,290 results after de-duplication (1,958 detected automatically with Zotero, 60 manually identified, and 28 identified via Covidence^11^). Of those, 3,104 (94%) were excluded via title and abstract screening. If the abstract had only been partially available during title and abstract screening, then the complete abstract was added prior to the full-text review stage. An additional 25 were excluded based on these full abstracts.

Of the 186 (6%) papers that merited full-text review, ten were irretrievable and they were coded for bibliographic information only and not incorporated into the results below (they are included in the abstract-level database). Following full text review, 109 (62%) of the remaining 176 papers were excluded (see Fig. 1 for reasons), leaving 67 (38%) unique results from the bibliographic search.

The first author then manually reviewed the references cited by those 67 articles as well as by any on-topic review papers that had been excluded. This “backward” search identified five additional results, all of which were included. An additional 21 included articles were added from researcher-to-researcher channels (eight from emails to authors, seven from Twitter posts, and six from Listserv posts). After adding these 26 additional results to the 67 identified via bibliographic search, the final full-text systematic map included 93 articles (Appendix C) documenting a total of 181 studies. Researcher-to-research channels yielded two additional references for the abstract-level database, for a total of 12 (see https://osf.io/37xma/).

The only pre-existing research synthesis (Dechêne et al., 2010) included 25 results, 22 of which were among the 93 articles we had already identified. We were unable to obtain the three additional results that were based on unpublished data.

All 58 of the published articles included in the final map were written in English. Of the 35 unpublished references, two were undergraduate theses written in Spanish and one was a PhD thesis in German. The abstract, methods, and results sections of the Spanish theses were translated, and the German PhD included three manuscripts prepared for submission in English.

The full-text database for the 93 articles included in our review is available at https://osf.io/37xma/ and includes Google Scholar links for each article and citations counts from both Web of Science and Google Scholar (completed on 17 November 2020). As of 2 October 2020, none of the articles had been retracted.

All 93 articles were run through statcheck (Rife, Nuijten, & Epskamp, 2016) to check for errors in statistical reporting. Statcheck recomputes p-values and compares them to those reported in the text. Inconsistent p-values are recorded as an “error.” If the reported result was significant and the recomputed result was not, or vice versa, the result was recorded as a “decision error.” Of the 57 PDFs that were readable, only 31 had no issues, 26 contained errors, and four of those were decision errors (for a summary, see Appendix D; for complete statcheck reports, see https://osf.io/r3cwg/). However, no errors related to the p-values for the overall effect of illusory truth: For all studies, the value that statcheck recalculated for the critical test matched the one reported in the paper. As preregistered, we did not further evaluate the statcheck results.

### Systematic map findings

The Stage 1 manuscript was preregistered and is available at https://osf.io/ar4hm. Deviations from the accepted Stage 1 are explicitly documented at https://osf.io/2hcyr/. We coded a total of 74 variables for each article (see Table 2; full coding of all variables along with coding criteria/instructions are available at https://osf.io/a9mfq/). Here we report the variables likely to be of broad interest and most relevant for identifying gaps in the literature. Despite best efforts to avoid error, as with any project of this scale, coding errors may occur. We will maintain an updated version of all tables/figures and the associated database at https://osf.io/dm9yx/, and will document any errors, corrections, or comments we receive.

#### Range of publication types, countries, and experimental aims

In order to understand the breadth of research conducted on the illusory truth effect, in this section we evaluate the range of article types, publication locations and dates, and the overarching aims of the included studies. Table 3 categorizes the types of documents included in the map.

**Table 3:** Types of sources included in the systematic map by publication status

Article type	N
Published	
Peer-reviewed journal article	57
Book chapter	1
Unpublished	
PhD thesis	8
Summary	8
Article	5
Preprint	5
MSc dissertation	4
Conference paper	3
UG dissertation	2

**Fig. 2:**
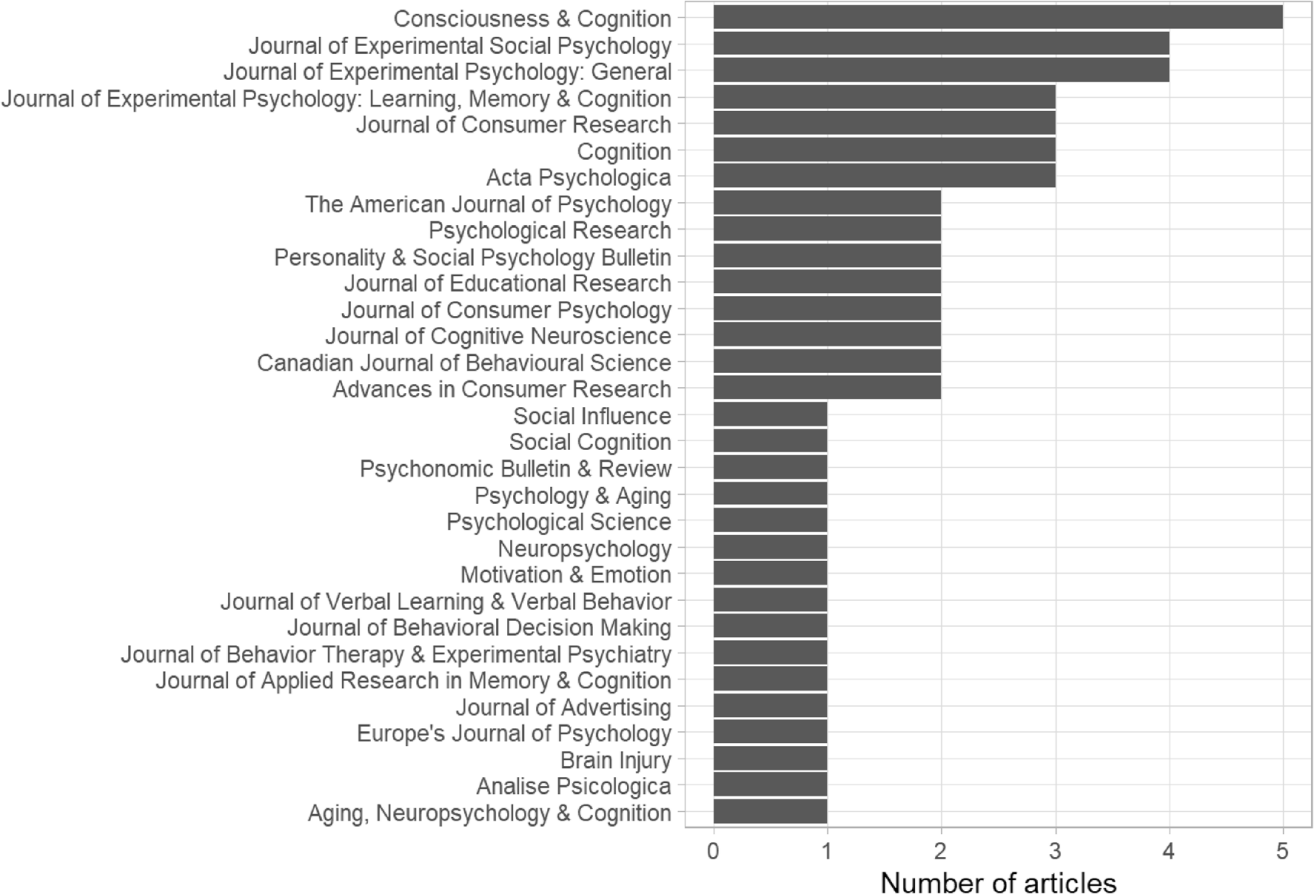
Journals that have published the illusory truth effect articles included in the map

The majority of published articles appeared in psychology journals, followed by marketing, neuroscience, and education journals (see Fig. 2). We used www.openaccessbutton.org to check whether the 58 published works were available on open access. Seventeen were on open access and the remaining 41 were behind a paywall.

**Fig. 3:**
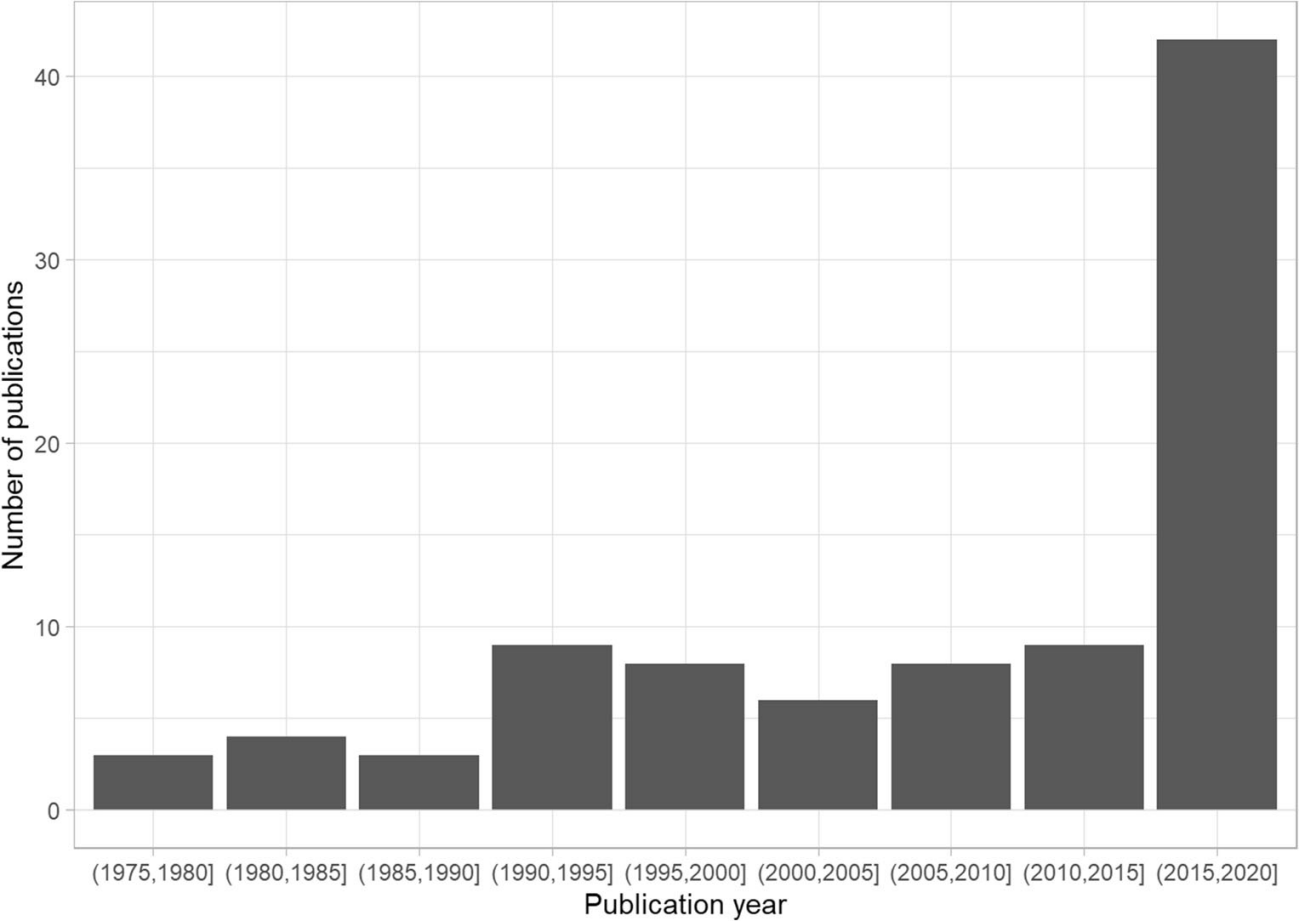
Date of publication/completion articles included in the systematic map. The figure includes both published and unpublished studies. The square bracket means inclusive and the parentheses means exclusive (e.g., the range (1975, 1980] excludes 1975 but includes 1980)

Since the first paper on the illusory truth effect was published in 1977 (Hasher, Goldstein, & Toppino, 1977), there has been a general upward trend in research on the topic (see Fig. 3), with an increase since 2015 (2016: seven papers; 2017: six papers; 2018: six papers; 2019: eight papers; 2020: 15 papers^12^). Of the 93 papers in our map, 54 appeared in 2010 or later.

**Fig. 4:**
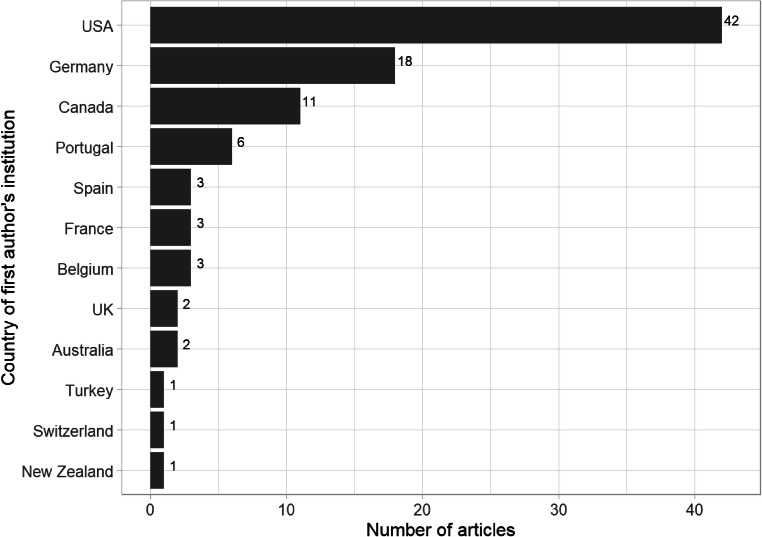
Number of articles included in the systematic map ordered by the country of the first author’s institution

Based on the first author’s institutional location, all published studies were conducted in 12 Western countries, with nearly half conducted in the USA (see Fig. 4). The lack of any studies from researchers in Asia, Africa, or Latin America appears to be a notable gap in the illusory truth effect literature. Although our exclusive use of English language search terms might have resulted in a sampling bias that missed work by authors from those regions, the vast majority of psychology literature is written in English. This gap warrants further investigation. If there are differences in the illusory truth effect based on culture or other global regional differences, the results in our systematic map cannot inform us about them.

We aimed to code the primary purpose of each study to determine whether measuring the illusory truth effect was the main experimental goal, or whether the goal was to measure variation in the effect. We focused on the abstract to see whether the authors stated an explicit aim and corresponding results. Many studies (36 or 20%) did not specify a clear goal in the abstract. Just 46 (25%) studies described the results of an overall test of the illusory truth. This figure is not surprising given that the majority of studies address issues that assume an overall illusory truth effect exists, and instead focus on variation in other factors.

Many studies (69 or 38%) aimed to examine variations in the magnitude of the overall illusory truth (i.e., moderation or mediation), and 67 (37%) reported finding variation of some sort. However, many studies focused on variations for outcomes other than the overall illusory truth effect (43 or 24%).

#### Experimental design, materials, measures, and participants

This section evaluates the types of participant groups tested, and the range of conditions and materials used in order to assess the level of standardization of experimental designs and the generalizability of the effect.

**Fig. 5:**
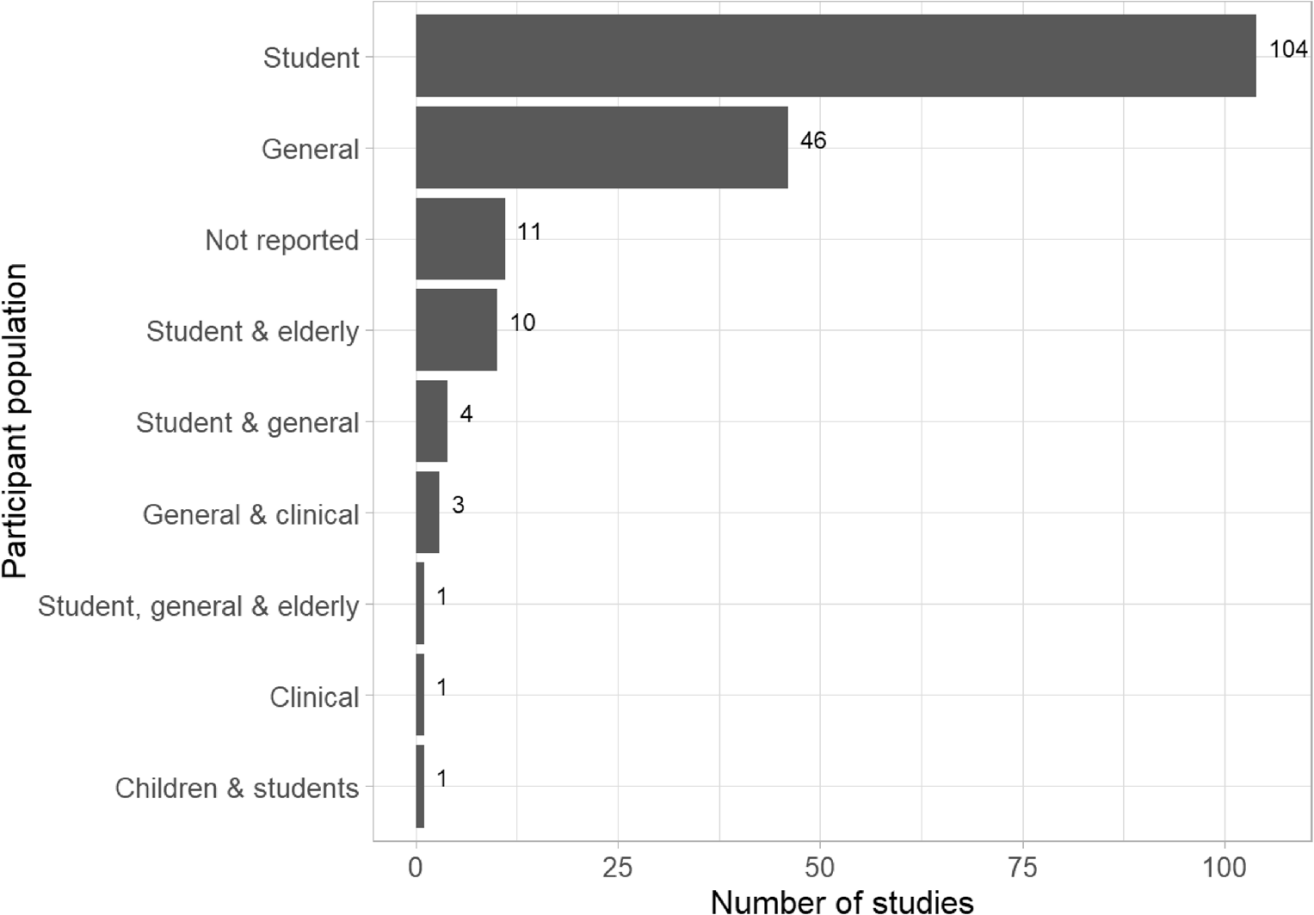
Frequency and variety of participant populations within the included studies

More than half of all studies used a student population (see Fig. 5). There was minimal research on harder to reach groups such as clinical populations and younger and older participants, revealing a gap in the knowledge base about the nature of the illusory truth effect in children and older adults.

Most studies were conducted in a lab or classroom (116, 64%), followed by online (48 or 27%). Two studies (1%) were conducted in participants’ homes, which might represent a more naturalistic, generalizable context in which to measure the effect. Eleven studies (6%) did not report the setting, two studies (1%) used various settings, and we lacked information for two studies (1%).

**Fig. 6:**
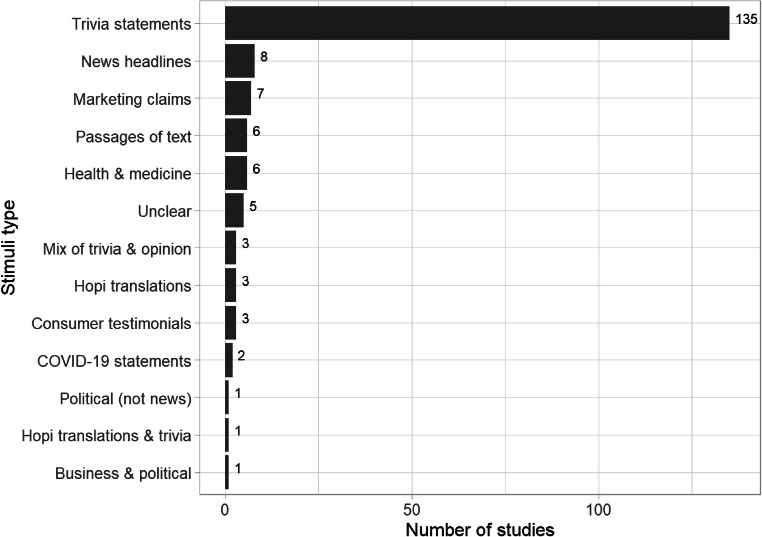
Frequency and variety of experimental stimuli within the included studies

Studies within the map overwhelmingly used trivia statements (135 or 75%) as the experimental stimuli (see Fig. 6). This finding highlights a gap in the evidence that may affect the generalizability of the effect. What research there is beyond trivia statements suggests that the illusory truth effect occurs using a variety of other stimuli including statements about health, news headlines, and politics. Given the importance of such topics, future research should focus on these areas and other topics relating to deeply held beliefs (e.g., beliefs about climate change).

Few studies (15 or 8%) tested whether participants already knew the truth/falsity of the experimental stimuli. If participants already know the answers to some trivia questions, they may use their existing knowledge when judging truth, thereby diminishing the effect of repetition (although prior knowledge does not provide total protection from the effect, see Fazio, 2020). Using normed trivia statements does not completely avoid this issue: Participants correctly answered 36% of the “unknown” statements from a normed set (Fazio, 2020). Disentangling prior knowledge from the effect of repetition looks to be an interesting direction for future studies.

**Fig. 7:**
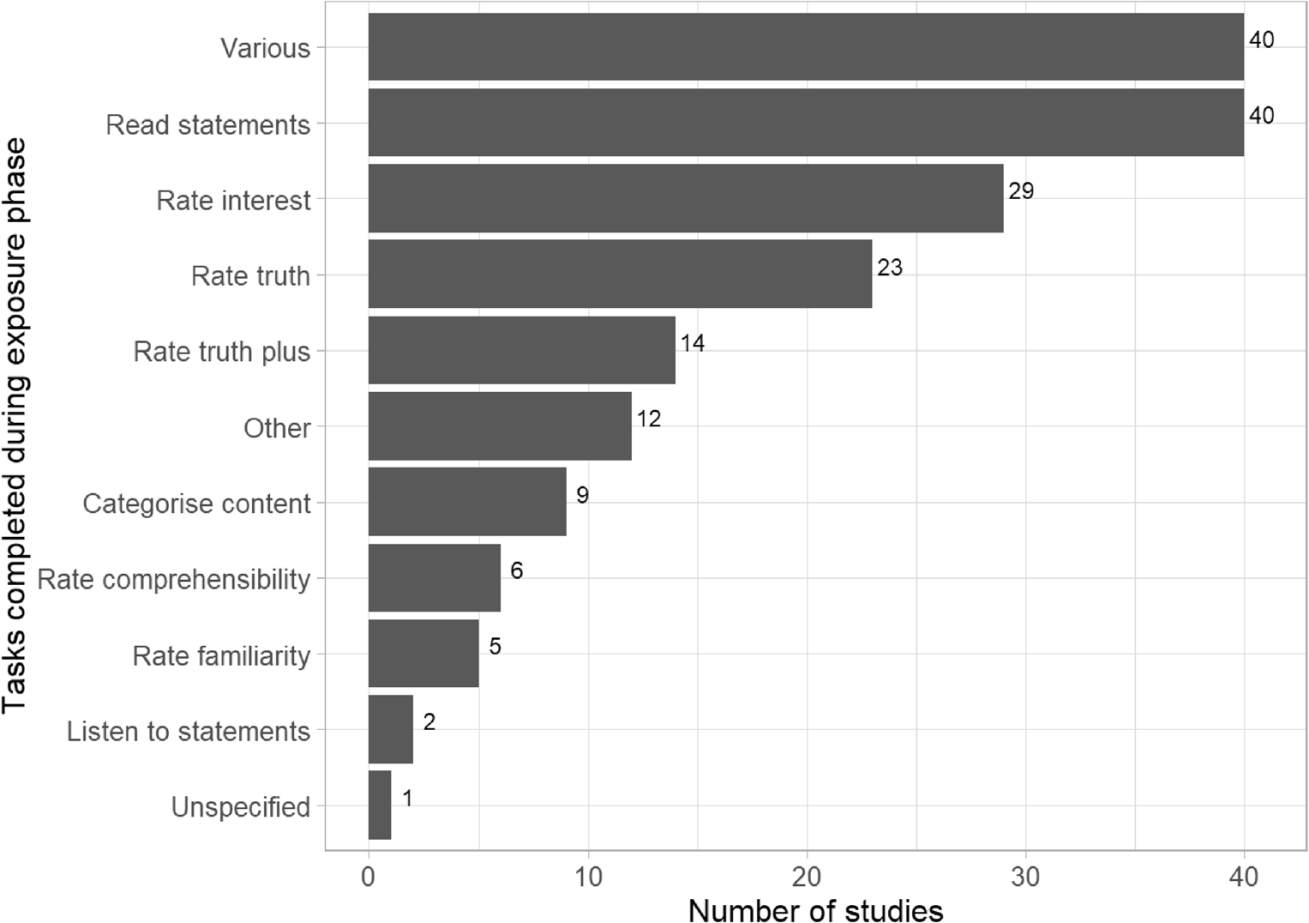
Frequency and range of tasks completed during the exposure phase. If the task involved rating truth and another task, they were coded as “rate truth plus.” Other combinations of two or more tasks were coded as “various.” All tasks involved reading or listening to the critical stimuli. If participants did not carry out any additional task with the critical stimuli, they were coded as “read statements” or “listen to statements”

We coded 55 different tasks or combinations of tasks carried out with the experimental stimuli during the exposure phase (we grouped tasks into meaningful clusters for the purposes of reporting; Fig. 7). This level of task variability shows a lack of standardized method for testing the illusory truth effect. Furthermore, some tasks could affect participants’ ratings during the test phase. For example, evaluating stimuli might result in a different level of processing compared to just reading or hearing them (42 or 23%). Asking participants to rate their interest in the stimuli (29 or 16%) could imply that the statements are true and might inadvertently tap into processes that are similar to explicit truth judgements. Similarly, 37 (21%) studies required participants to give truth judgements during the exposure phase, which could encourage them to give consistent ratings during the test phase (Nadarevic & Erdfelder, 2014). Some studies that directly manipulate the exposure task have found that the choice of task moderates the effect. For example, participants rating interest (Brashier, Eliseev, & Marsh, 2020) or categorizing statements (Nadarevic & Erdfelder, 2014) show the illusory truth effect, but those rating truth do not. Further synthesis of the literature could compare effect sizes as a function of exposure task, and this could be complemented by research in which the choice of task is systematically varied.

Similarly, there was no consistency in the filler tasks used during the retention interval between exposure and test. Fifty-eight different tasks or combinations of tasks were reported, ranging from demographics questions to number puzzles to personality questionnaires. As with the exposure task, it is possible that different filler tasks could influence the subsequent test phase. Sixty-nine (38%) studies did not specify the filler task, meaning that these studies cannot be evaluated for the influence of filler task on the effect.

**Fig. 8.**
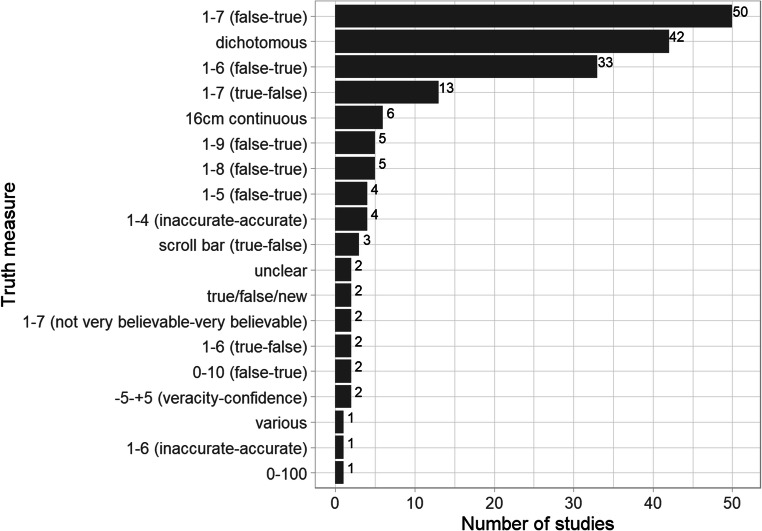
Frequency and variety of truth measures within the included studies. We lacked information for one study, k = 180

There was also great heterogeneity in the measures used to rate truth. Nineteen “truth” measures were coded in the map, including continuous scales from 1–100, Likert-type scales with and without neutral points, and dichotomous judgements (Fig. 8). In some cases, the truth measure varied within a paper without explanation. Measuring truth judgements in such diverse ways implies an underlying, latent truth continuum that can be measured in a binary or continuous way, yet there has been no validation or latent construct analysis in the literature. Given the quantity of evidence available, this area merits further synthesis to investigate whether the illusory truth effect differs as a function of the way in which truth judgements are measured. Additionally, future experimental research should systematically vary the measure to investigate illusory truth as a function of truth measure. Based on the homogeneity of research questions being asked (i.e., does repetition affect truth?), the variability in approaches to measuring truth seems worth addressing. Ideally, the field could establish a few reliable, validated measures and use them consistently (or provide justification for using alternative measures).

**Fig. 9.**
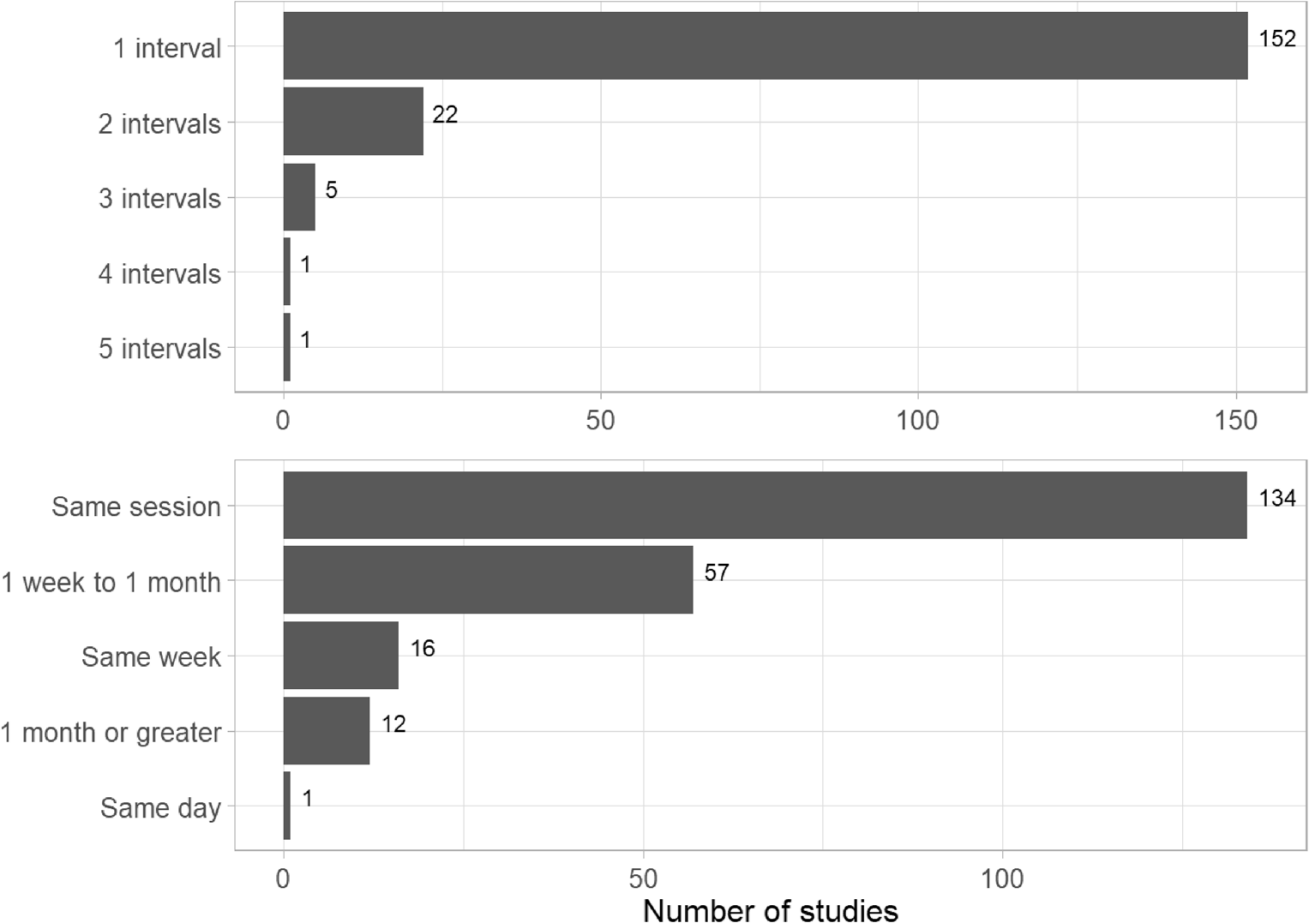
Top panel shows the number of retention intervals used in the 181 included studies. The bottom panel shows the length of the retention interval (i.e., time between exposure phase and test phase). Some studies used multiple retention intervals, n = 220.

In order to understand the illusory truth effect over time we need a range of retention intervals as well as studies that systematically track the effect over time using multiple retention intervals between exposure and test. We coded the length of the retention interval and the number of intervals used by each study. Overall, the vast majority of studies used a single retention interval, in most studies, the test phase was conducted in the same session as the exposure phase^13^ (see Figure 9). Relatively few studies used multiple testing intervals, and all but 12 (6%) of the test stages occurred within one month of exposure. The literature includes almost no studies testing long intersession intervals, examining the effect over time, or exploring the temporal boundaries of the effect.

**Fig. 10.**
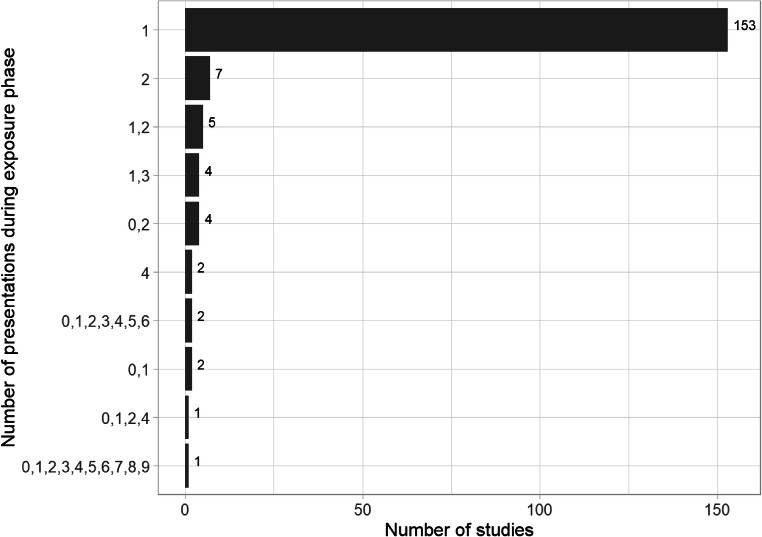
Number of presentations of experimental stimuli during the exposure phase within the included studies. For example, “1, 3” represents studies where individual stimuli were presented either 1 time or 3 times during the exposure phase.

Although many studies are motivated by the idea that repetition over time increases judged truth, relatively few studies varied the number of repetitions. At exposure phase the vast majority of studies (153 or 85%) presented the stimuli just once (see Figure 10). At test phase, almost all studies (167 or 92%) used a single session and presented participants with one exposure to the experimental stimuli (173 or 96%). Consequently the majority of studies are based on one presentation during the exposure and one during the test phase. Other combinations are repetitions are less studied, highlighting the need for studies that vary both the number of repetitions and the gaps between them to examine the illusory truth effect as it might occur in the real-world.

Likewise, although most studies used verbatim repetition of stimuli (148 or 82%), exact repetition in the real world is relatively rare. Gist repetition (8 or 4%) is likely to be more representative of real life information acquisition where repetitions can occur multiple times from multiple sources with variations in prose. For real-world generality we need further research based on repetitions of content, rather than repetitions of exact wording.

#### Openness, transparency, reproducibility and completeness of reporting

In this section we evaluate the completeness of reporting within the evidence base, the frequency of “positive” results, and various transparency practices, in order to assess whether the studies provide enough information to verify that they are reproducible and robust.

#### Completeness of reporting

**Fig. 11.**
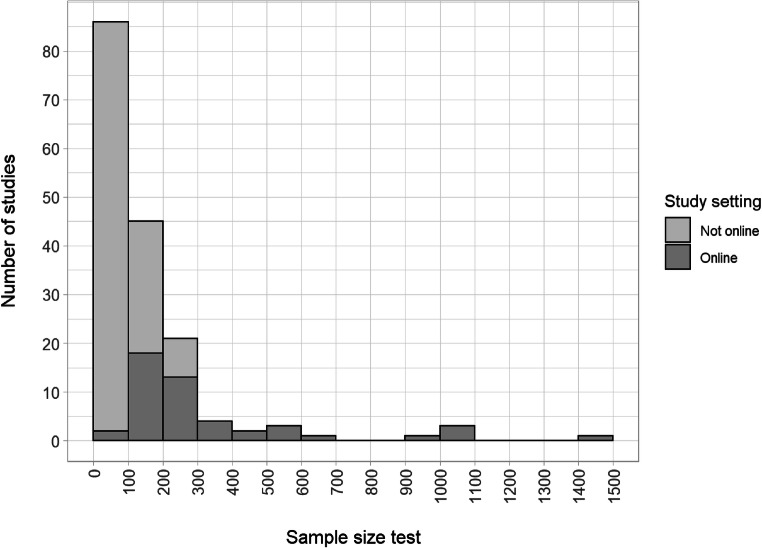
Sample sizes at test of included studies, split by whether studies were conducted online or not. We lacked information for three studies, k = 178. Note that the analysed sample sizes may have been smaller if data were excluded.

Transparent and complete reporting of sample size should include an explanation of the sample size selected and details of any data dropped from analyses. Study sample size ranged from 12 to 1478 (*M* = 153, *SD* = 196; see Figure 11), with online studies (*M* = 331) being larger than lab or classroom studies (*M* = 89). The majority of studies (139 or 77%) did not provide any rationale for the sample size selected. Only twenty-five (14%) provided a justification that included formal characteristics such as effect size or power level. Around half the studies (94 or 52%) analysed the data from all participants tested, and 68 studies reported exclusions^14^. But 14 (8%) studies had unexplained discrepancies between the reported and analysed sample sizes, suggesting unreported exclusions or possible errors.

Conducting a meta-analysis requires reported effect sizes or the descriptive statistics necessary to calculate them. Around three quarters of studies (129 or 71%) reported the results of the overall illusory truth effect in the results section, and of those 74 (57%) reported the effect size. Just over half of studies (102 or 56%) reported the overall means for repeated versus new statements. In the remaining studies, the means were potentially calculable from information provided (51 or 28%), or the information was not reported (23 or 13%). Only 47 (26%) studies reported the variance or SD for the critical means, 40 (22%) gave a range or provided some information that might make it possible to calculate the variability, but 89 (49%) studies did not provide measures of variance or enough information to calculate them^15^. Based on this incomplete reporting, an accurate meta-analysis of the entire literature is not possible. However the database will allow researchers to identify meaningful groups of studies that might provide enough information to be meta-analysed.

#### Transparency and reproducibility

We coded open-science practices to assess the transparency and potential reproducibility of the literature. Note that if the authors reported using an open practice (e.g., sharing materials) and evidence of that practice was available (i.e., *some* materials were shared), we coded the study as using that open practice. We did not, however, verify that sufficient materials were shared to enable a replication attempt.

**Fig. 12:**
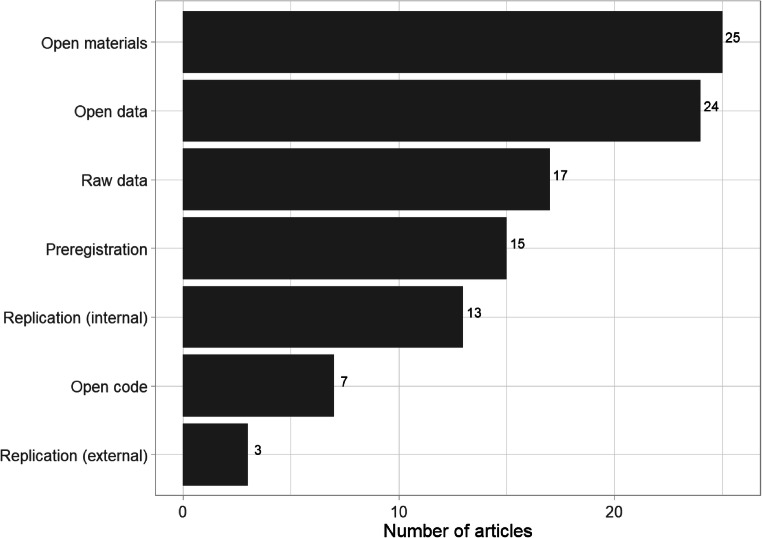
Frequency of open science practices used within the 93 included articles in the systematic map. If one study within a paper used that open practice it was coded as using that practice.

Most open practices were rare (see Figure 12). The most commonly used practice was sharing of materials, although this was largely driven by preprints and PhD theses. Only a small subset of papers reported sharing open data (24 or 26%), and we were able to access raw data for 17 (18%). Even fewer (7 or 8%) reported available analysis code, meaning that researchers interested in verifying the reproducibility of results could do so only for a minority of studies.

Fifteen (16%) papers reported a preregistered study, with seven of those appearing in 2020, indicating that preregistration is a new and possibly increasing practice in this literature. Although we did not carry out a comprehensive evaluation of those preregistration protocols, we note that several lacked comprehensive details about the procedures and analysis plans. As noted by others, a lack of detail is problematic because it does not sufficiently restrict researcher degrees of freedom (Bakker et al., 2020; Claesen, Gomes, Tuerlinckx, & Vanpaemel, 2019). That lack of precision might be particularly problematic for this literature given that the lack of methodological standardisation across the exposure task, filler task, and truth measures provides opportunities for researcher degrees of freedom. Preregistration represents an area for improvement: In addition to more preregistrations, the field needs more comprehensive preregistrations (or even better, Registered Reports) with sufficient detail to control type 1 error rates.

Thirteen articles (14%) described a built-in (“internal”) replication of a study reported in the same article, whereas only three (3%) included a replication of a study not reported in the same article (“external replication”). Replications are vital for verification, and replicability is a necessary condition for the accumulation of knowledge. The absence of independent replications, combined with the lack of available code and data, creates uncertainty about the robustness of the evidence in the literature. Further, our estimates of open science practices might overstate their commonality because we coded the article as using an open science practice even if not all of the studies reported in that article did so.

#### Publication bias

A literature without publication bias should include both positive and negative results. We coded the results of the overall illusory truth effect, as defined by the authors. Note that we have not evaluated the veracity of the claims about findings of the illusory truth effect. Nor have we formally assessed the magnitude of the reported effects (a future systematic review or meta-analysis could do so). Rather, we documented claims of having observed an illusory truth effect. Therefore these tallies should not be used to assess the presence or absence of an effect.

Of the 129 studies (71%) that reported inferential statistics for the overall illusory truth effect, 124 (96%) reported that the effect was either statistically significant or observed^16^. Surprisingly, the proportion of positive results was similar regardless of publication status or availability of open data. If all of these studies were testing real effects (not false positives), that means they averaged 96% power. However, within the psychological literature as a whole, power is estimated to be less than 50% (Cohen, 1990), and perhaps as low as 35% (Bakker, van Dijk, & Wicherts, 2012). We did not evaluate the power of the included studies, but given that sample sizes at test ranged from 12 to 1478, and only 25 studies reported some level of formal power analysis, it seems unlikely that all of these studies had ≥ 96% power (if they did, the effect sizes under investigation would have to vary massively as well, and the sample sizes for individual studies would have needed nearly perfect calibration with the true effect size under study). The high proportion of positive results might instead provide evidence of publication bias. The percentage of statistically significant (i.e., positive) results in this literature is similar to that reported for other literatures or for the field as a whole: 95.56% (Sterling et al., 1995), 91.5% (Fanelli, 2010) and most recently 96% (Scheel, Schijen, & Lakens, 2020). In contrast, a recent assessment of Registered Reports which should be comparatively bias free showed just 44% (Scheel et al., 2020).

## Discussion

### Key Findings

The aim of this map was to document the available evidence on the illusory truth effect. We identified 181 separate studies reported in 93 empirical articles, chapters, or theses. The research spans five decades, 12 countries, and is largely published in psychology journals. The literature includes many studies using verbatim repetition of trivia statements with student participants in a single session. It includes few studies that vary the number of repetitions or the persistence of the effect over time, tasks, and materials.

Overall, the majority of studies used fairly simple and quick data collection procedures that do not provide a strong test of the generality or practical importance of the illusory truth effect: Most studies did not look at the effects of delay, the effects of repeated exposures, or population differences. To increase the generalisability of the effect, future research should diversify beyond the frequently studied domains and focus on questions that help us understand how the effect might work in the real world, such as “how long lasting is the effect of single/multiple repetitions?”. The literature lacks the breadth of evidence to generalise beyond the commonly used participants groups and materials. Future research using carefully designed multi-lab studies, such as those conducted via the Psychological Science Accelerator, would be an appropriate way to ascertain the generalisability of findings in this literature.

In addition to using a restricted set of stimuli and populations, the experimental methodology was characterised by a lack of standardisation in the tasks and measures used to measure the illusory truth effect. There was large heterogeneity in the tasks used during the exposure phase and intersession interval, and there was substantial variability in the way in which truth judgements were measured. Work is needed both to investigate the potential effect of this variability on the magnitude of the illusory truth effect and to standardize measures in order to increase the reliability and validity of subsequent research. Future research should focus both on synthesizing the available evidence on these topics and on systematically varying these factors within preregistered experiments.

While open science practices are increasing, the lack of available raw data and code means that attempts to reproduce the research would only be possible in a small minority of cases. This factor, along with a dearth of close replication studies, few preregistrations, and largely absent justifications for sample sizes raise concerns about the credibility and robustness of the literature. A lack of sample size justification alone does not mean that the study had low power. However, many literatures appear to be dominated by studies with relatively low power (Bakker et al., 2012; Cohen, 1990), suggesting that researchers are (or were) unaware of the problem of low power. And, studies that do include a power analysis likely are conducted by researchers who recognize the need for larger sample sizes. Consequently, significant results from studies that justified their sample size might be more likely to reflect true positive findings than those that did not. Consistent with the idea, the mean sample size for studies in the map that reported any form of sample size justification was more than double (*M* = 277.4, *SD* = 305.1) the mean for studies that did not (*M* = 126.6, *SD* = 148.0).

This map identified high levels of positive results within the literature, signifying potential publication bias. In addition, the levels of incomplete reporting preclude a meta-analysis of the entire evidence base. There is no reason to believe that these issues are more or less severe in this literature than in other fields – most fields have publication bias. Regardless of their prevalence, these issues warrant attention and improvement. To assist future subgroup meta-analyses, we recommend that authors report full descriptive statistics for all measures as well as correlations among measures for repeated-measures designs. Ideally, all future research on the illusory truth effect will make raw data (and a codebook) available in a public repository such as the OSF.

### How to use this systematic map and database

This map illustrates the quantity and diversity of research on the illusory truth effect. Although we coded articles for open science practices, we did not carry out a critical appraisal. Therefore, a high prevalence of a particular type of evidence in this map indicates only that it has been studied frequently, and not that it has been studied well or that the evidence is strong. Further syntheses are required to make evaluations of effectiveness and effect size.

The map is accompanied by a database available at https://osf.io/37xma/. The database serves as a searchable resource on the illusory truth effect. This paper reports results that will be of general interest, but the database includes more information and makes it possible for researchers to filter based on specific variables of interest, to understand the areas that are well studied, which papers studied them, and where there is scope for further research.

Researchers may wish to conduct a meta-analysis on some subset of the literature. The systematic map database can be filtered based on specific areas of interest (e.g., studies that use health statements as stimuli) and codes #73 and #74 then can be used to identify whether means and measures of variance are reported for that subset of the literature. To progress from this map to a full systematic review is a relatively small task since much of the time-consuming aspects of the review, such as searching and screening, have already been completed. Before conducting a further review, we recommend that a full critical appraisal is completed as well as an update to include new evidence.

### Limitations of the systematic map

Although we used the R package litsearchr (Grames et al., 2019b) to reduce bias in, and increase the diversity of, our search term selection, we recognize that as a team of psychologists we may have missed terms used in adjacent fields. Additionally, due to resource constraints, all search terms were in English. Although the majority of psychological literature is written in English, there could be literature in other languages that our search terms did not identify. However, we have clearly and transparently reported our search methodology, so the map could be updated with further searches in multiple languages.

Coding the primary goal and results of each study from each article’s abstract was challenging due to unclear reporting. Whereas the majority of variables coded in this map were objective, these codes required more interpretation and may therefore be less reproducible. To help overcome this issue, the primary coder (ELH or SJW) sought a second opinion on these codes where necessary.

We coded the first author’s institutional location as a proxy for the location in which the study was conducted. This measure is likely to be accurate in most cases, but it is possible that some studies were conducted outside of the lead author’s home country.

When assessing open science practices, we coded a paper as having used a practice if there was any evidence of that open practice (e.g., a file containing data was shared). We did not evaluate whether the shared materials were complete or usable (e.g., whether they included relevant data, a codebook, or runable code), so we cannot be certain that they allow for reproducibility or that they would be sufficient for a replication. Equally, although we verified whether or not preregistration documents existed, we did not thoroughly review the details and the extent to which the procedures reported in the article matched those in the preregistration. Insufficiently detailed preregistrations might not adequately constrain researcher degrees of freedom and type 1 errors (Bakker et al., 2020; Claesen et al., 2019). In sum, our findings estimate the prevalence of open science practices but not whether those practices are working as intended.

### Future research summary

Throughout the paper we highlight knowledge gaps in the current literature on the illusory truth effect. We see three general directions for future research: First, test the generalizability of the effect by using more diverse stimuli, participants, intervals, and numbers of repetitions. Multi-lab Registered Reports would be an ideal mechanism for such research. Second, examine the dependency of the effect on the choice of exposure task and truth measure by synthesizing the current research. Last, increase the reliability of illusory truth research by standardizing the exposure task and establishing validated truth measures.

## Supplementary information

(PDF 884 kb)

## Footnotes

1 This figure likely also reflects HARKing, p-hacking, and other questionable research practices that can occur prior to article submission.

2 The results of the pilot coding are available at https://osf.io/jd72s/.

3 Although this paper is concerned with meta-analyses, the recommendations remain relevant for systematic mapping.

4 ROSES was developed for systematic reviews and maps in the field of conservation and environmental management, but can be applied in the current context without the need for adaptation.

5 We hope that others will continue to update the database.

6 We preregistered issuing a call through the Academy of Marketing Science (AMS). We attempted to contact AMS three times but received no response.

7 Review papers were searched as detailed under “Manual searches.”

8 The original second author of the Stage 1 Registered Report (FVT) withdrew from the project and was replaced by SJW. Thus, the second author was an external, independent coder who joined after Stage 1 IPA had been received.

9 We did not compare highlighting when evaluating the reliability of the coding instructions.

10 Highlighted by the primary coder.

11 We did not preregister the use of manual or Covidence’s deduplication. However, both methods picked up duplicates that Zotero missed.

12 Note that electronic searches were conducted at the beginning of February 2020.

13 For the purposes of reporting we re-coded each idiosyncratic interval into the following categories: same session, same day, same week, 1 week to 1 month, 1 month or greater. Within the map database we coded each retention interval as reported in the paper (e.g., 2 minutes, 2 hours, etc). For the 29 studies that used two or more retention intervals, we coded each interval separately and thus have 220 intervals from the 181 studies.

14 We lacked this information for five studies (3%).

15 We lacked this information for five studies (3%).

16 Fifty two studies (29%) did not report the results of an overall illusory truth effect. It is possible that in some cases this was due to the result being non-significant. However without a preregistration, we do not know their a priori aims and whether they planned to analyse the overall effect.

## Declarations

### Stakeholder engagement

There are no stakeholders involved in this systematic map other than the research team and reviewers of the stage 1 Registered Report.

## References

[CR1] Allaire, J., Xie, Y., McPherson, J., Luraschi, J., Ushey, K., Atkins, A., … Iannone, R. (2020). *rmarkdown: Dynamic Documents for R*. Computer software. Retrieved from https://github.com/rstudio/rmarkdown

[CR2] Arkes HR, Hackett C, Boehm L (1989). The generality of the relation between familiarity and judged validity. Journal of Behavioral Decision Making.

[CR3] Bakker M, van Dijk A, Wicherts JM (2012). The rules of the game called psychological science. Perspectives on Psychological Science.

[CR4] Bakker M, Veldkamp CLS, van Assen MALM, Crompvoets EAV, Ong HH, Nosek BA (2020). Ensuring the quality and specificity of preregistrations. PLoS Biology.

[CR5] Brashier NM, Eliseev ED, Marsh EJ (2020). An initial accuracy focus prevents illusory truth. Cognition.

[CR6] Brown AS, Nix LA (1996). Turning lies into truths: Referential validation of falsehoods. Journal of Experimental Psychology: Learning, Memory, and Cognition.

[CR7] Claesen, A., Gomes, S. L. B. T., Tuerlinckx, F., & Vanpaemel, W. (2019). Preregistration: comparing dream to reality. 10.31234/osf.io/d8wex 10.1098/rsos.211037 PMC8548785 34729209

[CR8] Cohen J (1990). Things I have learned (so far). American Psychologist.

[CR9] Corker, K. S. (2018). Strengths and weaknesses of meta-analyses. PsyArXiv. 10.31234/osf.io/6gcnm

[CR10] Dechêne A, Stahl C, Hansen J, Wänke M (2010). The truth about the truth: A meta-analytic review of the truth effect. Personality and Social Psychology Review.

[CR11] Dreyfuss, E. (2017). Want to make a lie seem true? Say it again. And again. And again. Retrieved December 18, 2018, from https://www.wired.com/2017/02/dont-believe-lies-just-people-repeat/

[CR12] Fanelli D (2010). “Positive” results increase down the Hierarchy of the Sciences. Plos One.

[CR13] Fazio, L. K. (2020). Repetition increases perceived truth even for known falsehoods. PsyArXiv. 10.31234/osf.io/2u53a

[CR14] Fazio LK, Brashier NM, Payne BK, Marsh EJ (2015). Knowledge does not protect against illusory truth. Journal of Experimental Psychology: General.

[CR15] Fazio, L. K., Rand, D. G., & Pennycook, G. (2019). Repetition increases perceived truth equally for plausible and implausible statements. PsyArXiv. 10.31234/osf.io/qys7d 10.3758/s13423-019-01651-4 31420808

[CR16] Garcia-Marques T, Silva RR, Mello J (2017). Asking simultaneously about truth and familiarity may disrupt truth effects. Análise Psicológica.

[CR17] Gigerenzer G (1984). External validity of laboratory experiments: The frequency-validity relationship. The American Journal of Psychology.

[CR18] Grames EM, Stillman AN, Tingley MW, Elphick CS (2019). An automated approach to identifying search terms for systematic reviews using keyword co-occurrence networks. Methods in Ecology and Evolution.

[CR19] Grames, E. M., Stillman, A., Tingley, M., & Elphick, C. (2019b). litsearchr: Automated search term selection and search strategy for systematic reviews [Computer software]. Downloaded from https://elizagrames.github.io/litsearchr/

[CR20] Haddaway NR (2018). Open Synthesis: On the need for evidence synthesis to embrace Open Science. Environmental Evidence.

[CR21] Haddaway NR, Collins AM, Coughlin D, Kirk S (2015). The role of google scholar in evidence reviews and its applicability to grey literature searching. Plos One.

[CR22] Haddaway NR, Feierman A, Grainger MJ, Gray CT, Tanriver-Ayder E, Dhaubanjar S, Westgate MJ (2019). EviAtlas: A tool for visualising evidence synthesis databases. Environmental Evidence.

[CR23] Haddaway, N. R., Macura, B., Whaley, P., & Pullin, A. (2018a). ROSES Flow Diagram for Systematic Maps. Version 1.0. *Figshare*. 10.6084/m9.figshare.6085940

[CR24] Haddaway NR, Macura B, Whaley P, Pullin AS (2018). ROSES RepOrting standards for Systematic Evidence Syntheses: Pro forma, flow-diagram and descriptive summary of the plan and conduct of environmental systematic reviews and systematic maps. Environmental Evidence.

[CR25] Hardwicke TE, Wallach JD, Kidwell MC, Bendixen T, Crüwell S, Ioannidis JPA (2020). An empirical assessment of transparency and reproducibility-related research practices in the social sciences (2014–2017). Royal Society Open Science.

[CR26] Harzing, A. W. (2007). Publish or Perish. Retrieved April 24, 2019, from https://harzing.com/resources/publish-or-perish

[CR27] Hasher L, Goldstein D, Toppino T (1977). Frequency and the conference of referential validity. Journal of Verbal Learning and Verbal Behavior.

[CR28] Huxley A (1932). Brave new world.

[CR29] James KL, Randall NP, Haddaway NR (2016). A methodology for systematic mapping in environmental sciences. Environmental Evidence.

[CR30] Lakens D, Hilgard J, Staaks J (2016). On the reproducibility of meta-analyses: six practical recommendations. BMC Psychology.

[CR31] Lewandowsky S, Stritzke WGK, Oberauer K, Morales M (2005). Memory for fact, fiction, and misinformation: the Iraq War 2003. Psychological Science.

[CR32] Moher D, Shamseer L, Clarke M, Ghersi D, Liberati A, Petticrew M (2015). Preferred reporting items for systematic review and meta-analysis protocols (PRISMA-P) 2015 statement. Systematic Reviews.

[CR33] Munafò MR, Nosek BA, Bishop DVM, Button KS, Chambers CD, du Sert NP (2017). A manifesto for reproducible science. Nature Human Behaviour.

[CR34] Nadarevic L, Erdfelder E (2014). Initial judgment task and delay of the final validity-rating task moderate the truth effect. Consciousness and Cognition.

[CR35] Nosek BA, Alter G, Banks GC, Borsboom D, Bowman SD, Breckler SJ (2015). Promoting an open research culture. Science.

[CR36] Paschal, O. (2018). Trump’s Tweets and the Creation of ‘Illusory Truth. Retrieved December 11, 2018, from https://www.theatlantic.com/politics/archive/2018/08/how-trumps-witch-hunt-tweets-create-an-illusory-truth/566693/

[CR37] Paul, C., & Matthew, M. (2016). *The Russian “firehose of falsehood” propaganda model* (pp. 2–7). Rand Corporation.

[CR38] Pennycook G, Cannon TD, Rand DG (2018). Prior exposure increases perceived accuracy of fake news. Journal of Experimental Psychology: General.

[CR39] Polage DC (2012). Making up history: False memories of fake news stories. Europe’s Journal of Psychology.

[CR40] Core Team R (2019). *R: A Language and Environment for Statistical Computing*. Computer software.

[CR41] Renkewitz F, Keiner M (2019). How to detect publication bias in psychological research. Zeitschrift für Psychologie.

[CR42] Resnick, B. (2017). The science behind why fake news is so hard to wipe out - Vox. Retrieved October 24, 2017, from https://www.vox.com/science-and-health/2017/10/5/16410912/illusory-truth-fake-news-las-vegas-google-facebook

[CR43] Rethlefsen, M. L., Kirtley, S., Waffenschmidt, S., Ayala, A. P., Moher, D., Page, M. J., … PRISMA-S Group. (2021). PRISMA-S: an extension to the PRISMA Statement for Reporting Literature Searches in Systematic Reviews. *Systematic Reviews*, *10*(1), 39. 10.1186/s13643-020-01542-z 10.5195/jmla.2021.962 PMC8270366 34285662

[CR44] Rife, S. C., Nuijten, M. B., & Epskamp, S. (2016). statcheck: Extract statistics from articles and recompute p-values [web application]. Retrieved from http://statcheck.io

[CR45] Roggeveen AL, Johar GV (2002). Perceived source variability versus familiarity: testing competing explanations for the truth effect. Journal of Consumer Psychology.

[CR46] Scheel, A. M., Schijen, M., & Lakens, D. (2020). An excess of positive results: Comparing the standard Psychology literature with Registered Reports. 10.31234/osf.io/p6e9c

[CR47] Shamseer L, Moher D, Clarke M, Ghersi D, Liberati A, Petticrew M (2015). Preferred reporting items for systematic review and meta-analysis protocols (PRISMA-P) 2015: elaboration and explanation. BMJ (Clinical Research Ed.).

[CR48] Sterling TD, Rosenbaum WL, Weinkam JJ (1995). Publication decisions revisited: the effect of the outcome of statistical tests on the decision to publish and vice versa. The American Statistician.

[CR49] Unkelbach C, Greifeneder R (2018). Experiential fluency and declarative advice jointly inform judgments of truth. Journal of Experimental Social Psychology.

[CR50] Unkelbach C, Rom SC (2017). A referential theory of the repetition-induced truth effect. Cognition.

[CR51] Wickham, H. (2011). The Split-Apply-Combine Strategy for Data Analysis. *Journal of Statistical Software*, *40*(1). 10.18637/jss.v040.i01

[CR52] Wickham, H., Averick, M., Bryan, J., Chang, W., McGowan, L., François, R., … Yutani, H. (2017). Welcome to the tidyverse. *The Journal of Open Source Software*. 10.21105/joss.01686

