# Supplementary material for: A reproducible systematic map of research on the illusory truth effect
Source: Psychon Bull Rev. 2021 Oct 27;29(3):1065–88. doi: 10.3758/s13423-021-01995-w (PMC9166874; doi:10.3758/s13423-021-01995-w)
Supplement: Supplementary file 1 — (PDF 884 kb) [file 13423_2021_1995_MOESM1_ESM.pdf]

Appendix A
Benchmark List

1. Begg, I., Anas, A., & Farinacci, S. *(1992). Dissociation of process*es in belief: Source recollection, statement familiarity, and the illusion of truth. Journal of Experimental Psychology: General, 121, 446-458.

2. Brown, A. S., & Nix, L. A. (1996). Turning lies into truths: Referential validation of falsehoods. Journal of Experimental Psychology: Learning, Memory*, and Cognition, 22, 1088-1100.*

3. Dechêne, A., Stahl, C., Hansen, J., & Wänke, M. (2009). Mix Me a list: Context moderates the truth effect and the mere exposure effect. Journal of Experimental Social Psychology, 45, 1117-1122.

4. Dechêne, A., Stahl, C., Hansen, J., & Wänke, M. (2010). The truth about the truth: A meta-analytic review of the truth effect. Personality and Social Psychology Review, 14, 238-257.

5. Fazio, L. K., Brashier, N. M., Payne, B. K., & Marsh, E. J. (2015). Knowledge does not protect against illusory truth. Journal of Experimental Psychology: General, 144, 993-1002.

6. Garcia-Marques, T., Silva, R. R., & Mello, J. (2017). Asking simultaneously about truth and familiarity may disrupt truth effects. Análise Psicológica, 35, 61-71.

7. Hawkins, S. A., & Hoch, S. J. (1992). Low-involvement learning: Memory without evaluation. Journal of Consumer Research, 19, 212-225.

8. Hawkins, S. A., Hoch, S. J., & Meyers Levy, J. (2001). Low-involvement learning: Repetition and coherence in familiarity and belief. Journal of Consumer Psychology, 11, 1-11.

9. Law, S., Hawkins, S. A., & Craik, F. I. M. (1998). Repetition-induced belief in the elderly: Rehabilitating age-related memory deficits. Journal of Consumer Research, 25, 91-107.

10. Mitchell, J. P., Dodson, C. S., & Schacter, D. L. (2005). fMRI evidence for the role of recollection in suppressing misattribution errors: The illusory truth effect. Journal of Cognitive Neuroscience, 17, 800-810.

11. Mitchell, J. P., Sullivan, A. L., Schacter, D. L., & Budson, A. E. (2006). Misattribution errors in Alzheimer's disease: The illusory truth effect. Neuropsychology, 20,185-192.

12. Mutter, S. A., Lindsey, S. E., & Pliske, R. M. (1995). Aging and credibility judgment. Aging and Cognition, 2, 89-107.

13. Nadarevic, L., & Erdfelder, E. (2014). Initial judgment task and delay of the final validity-rating task moderate the truth effect. Consciousness and Cognition, 23, 74-84.

14. Nadarevic, L., Plier, S., Thielmann, I., & Darancó, S. (2018). Foreign language reduces the longevity of the repetition-based truth effect. Acta psychologica, 191, 149-159.

15. Pennycook, G., Cannon, T. D., & Rand, D. G. (2018). Prior exposure increases perceived accuracy of fake news. Journal of Experimental Psychology: General, 147, 1865–1880.

16. Roggeveen, A. L., & Johar, G. V. (2002). Perceived source variability versus familiarity: Testing competing explanations for the truth effect. Journal of Consumer Psychology, 12, 81-91.

17. Scholl, S. G., Greifeneder, R., & Bless, H. (2014). When fluency signals truth: Prior successful reliance on fluency moderates the impact of fluency on truth judgments. Journal of Behavioral Decision Making, 27, 268-280.

18. Schwartz, M. (1982). Repetition and rated truth value of statements. American Journal of Psychology, 95, 393-407.

19. Silva, R. R., Garcia-Marques, T., & Mello, J. (2016). The differential effects of fluency due to repetition and fluency due to color contrast on judgments of truth. Psychological Research, 80, 821-837.

20. Unkelbach, C. (2007). Reversing the truth effect: Learning the interpretation of processing fluency in judgments of truth. Journal of Experimental Psychology: Learning, Memory, and Cognition, 33, 219-230.

Appendix B
Bibliographic Database and Grey Literature Searches

Bibliographic database searches were conducted with “apply equivalent subjects/map term to subject heading” de-selected.

Business Source Premier
Field: “Abstract or author-supplied abstract”
Using “Advanced Search”
Search string: ("illusory truth" OR "illusion* of truth" OR "induced truth effect" OR "reiteration effect" OR "tainted truth effect" OR “repetition based truth effect” OR “repetition induced increases” OR repeat OR repeated OR repeating OR repetition OR "prior exposure") AND AB (true* OR truth OR "truth effect*" OR belief) AND AB (statement* OR items OR stimulus OR stimuli OR claim* OR judgment* OR judgement* OR rating* OR "subjective truth" OR "truth value" OR “judged validity” OR “validity ratings” OR "processing fluency" OR "fluency effect*" OR "perceptual fluency")
Search modes - Boolean/Phrase

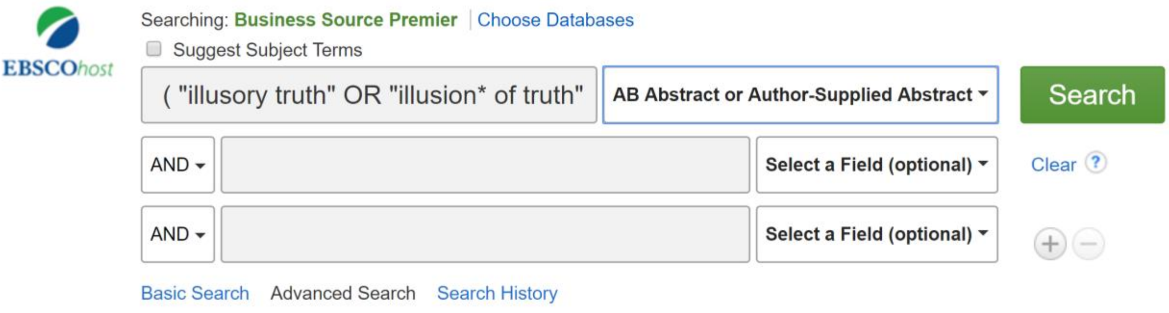

EconLit
Field: “Abstract”
Using “Advanced Search”
Search string: ("illusory truth" OR "illusion* of truth" OR "induced truth effect" OR "reiteration effect" OR "tainted truth effect" OR “repetition based truth effect” OR “repetition induced increases” OR repeat OR repeated OR repeating OR repetition OR "prior exposure") AND AB (true* OR truth OR "truth effect*" OR belief) AND AB (statement* OR items OR stimulus OR stimuli OR claim* OR judgment* OR judgement* OR rating* OR "subjective truth" OR "truth value" OR “judged validity” OR “validity ratings” OR "processing fluency" OR "fluency effect*" OR "perceptual fluency")
Search modes - Boolean/Phrase

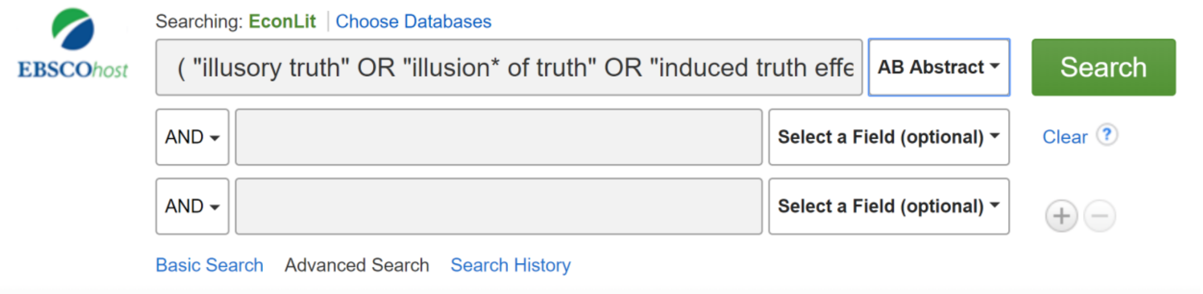

ERIC
Field: “Abstract”
Using “Advanced Search”
Search string: ("illusory truth" OR "illusion* of truth" OR "induced truth effect" OR "reiteration effect" OR "tainted truth effect" OR “repetition based truth effect” OR “repetition induced increases” OR repeat OR repeated OR repeating OR repetition OR "prior exposure") AND AB (true* OR truth OR "truth effect*" OR belief) AND AB (statement* OR items OR stimulus OR stimuli OR claim* OR judgment* OR judgement* OR rating* OR "subjective truth" OR "truth value" OR “judged validity” OR “validity ratings” OR "processing fluency" OR "fluency effect*" OR "perceptual fluency")
Search modes - Boolean/Phrase

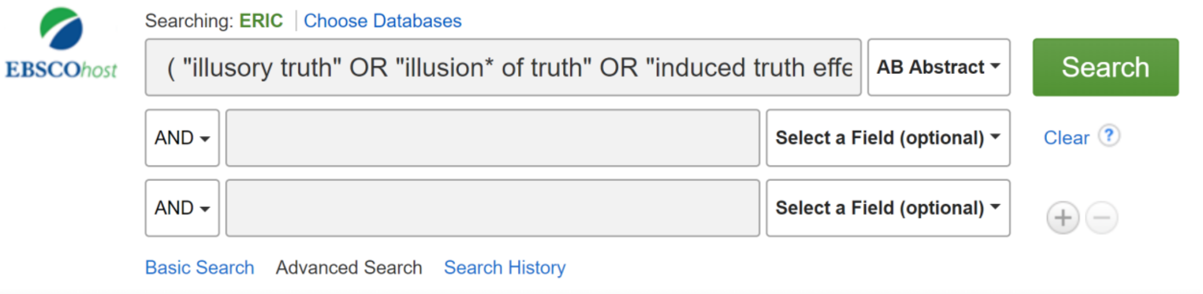

PsycINFO
Field: “Abstracts”
Using “Advanced Search”
Search string: (("illusory truth" OR "illusion* of truth" OR "induced truth effect" OR "reiteration effect" OR "tainted truth effect" OR "repetition based truth effect" OR "repetition induced increases" OR repeat OR repeated OR repeating OR repetition OR "prior exposure") AND (true* OR truth OR "truth effect*" OR belief) AND (statement* OR items OR stimulus OR stimuli OR claim* OR judgment* OR judgement* OR rating* OR "subjective truth" OR "truth value" OR "judged validity" OR "validity ratings" OR "processing fluency" OR "fluency effect*" OR "perceptual fluency"))

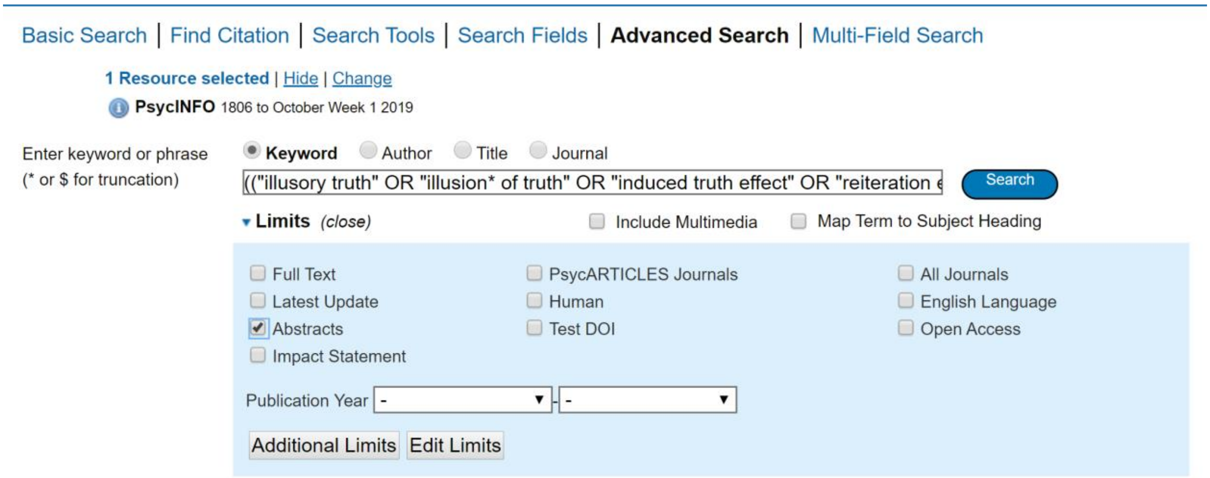

PubMed
Field: "Title/Abstract"
Using "Advanced Search Builder"
Search string: "illusory truth" OR "illusion* of truth" OR "induced truth effect" OR "reiteration effect" OR "tainted truth effect" OR "repetition based truth effect" OR "repetition induced increases" OR repeat OR repeated OR repeating OR repetition OR "prior exposure"
AND true* OR truth OR "truth effect*" OR belief
AND statement* OR items OR stimulus OR stimuli OR claim* OR judgment* OR judgement* OR rating* OR "subjective truth" OR "truth value" OR "judged validity" OR "validity ratings" OR "processing fluency" OR "fluency effect*" OR "perceptual fluency"

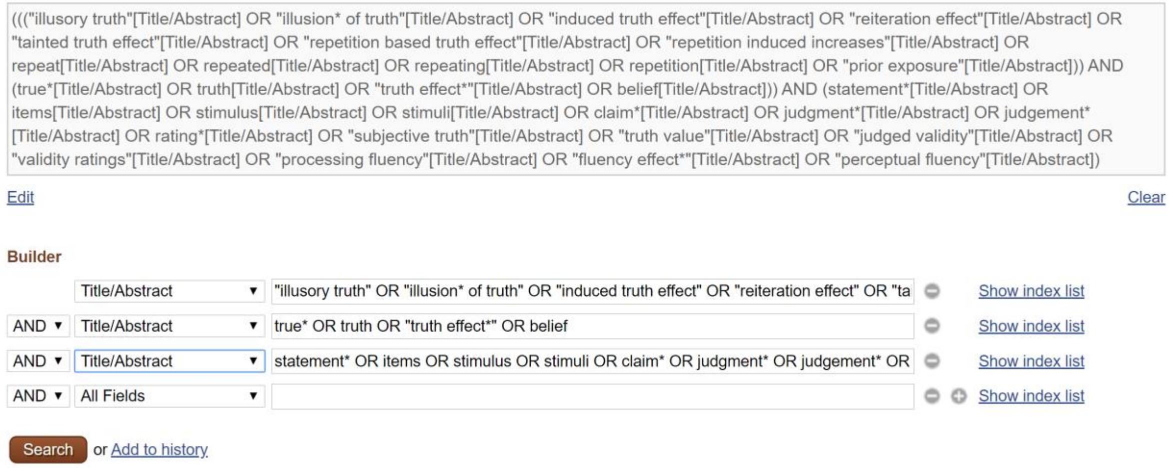

Scopus
Field: “Article title, Abstract, Keywords”
Using “Advanced Search”
Search string: TITLE-ABS-KEY(((("illusory truth" OR "illusion* of truth" OR "induced truth effect" OR "reiteration effect" OR "tainted truth effect" OR “repetition based truth effect” OR “repetition induced increases” OR repeat OR repeated OR repeating OR repetition OR "prior exposure") AND (true* OR truth OR "truth effect*" OR belief) AND (statement* OR items OR stimulus OR stimuli OR claim* OR judgment* OR judgement* OR rating* OR "subjective truth" OR "truth value" OR “judged validity” OR “validity ratings” OR "processing fluency" OR "fluency effect*" OR "perceptual fluency"))))

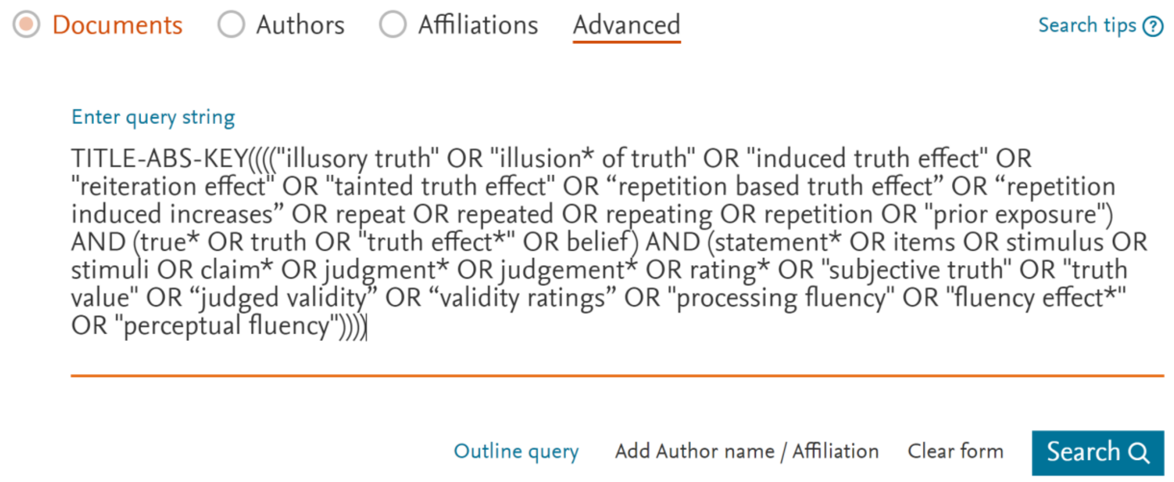

Web of Science
Field: “Topic”
Using “Basic Search”
Search string: (((("illusory truth" OR "illusion* of truth" OR "induced truth effect" OR "reiteration effect" OR "tainted truth effect" OR “repetition based truth effect” OR “repetition induced increases” OR repeat OR repeated OR repeating OR repetition OR "prior exposure") AND (true* OR truth OR "truth effect*" OR belief) AND (statement* OR items OR stimulus OR stimuli OR claim* OR judgment* OR judgement* OR rating* OR "subjective truth" OR "truth value" OR “judged validity” OR “validity ratings” OR "processing fluency" OR "fluency effect*" OR "perceptual fluency"))))
Timespan: All years. **Databases:** WOS, BCI, BIOSIS, KJD, MEDLINE, RSCI, SCIELO.
Search language=Auto

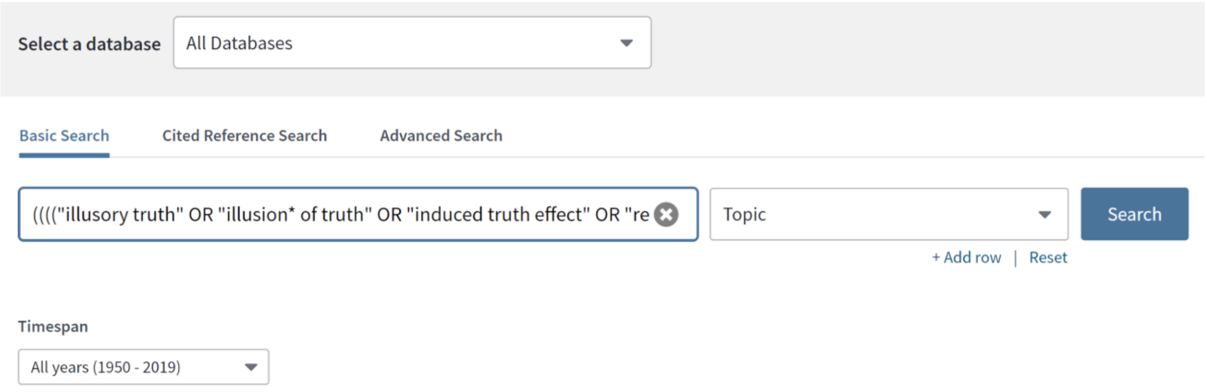

Google Scholar (via Harzing's Publish or Perish)

Google Scholar searches will be combined and deduplicated before being added to the master spreadsheet.

Search 1:

Title words "illusory truth"

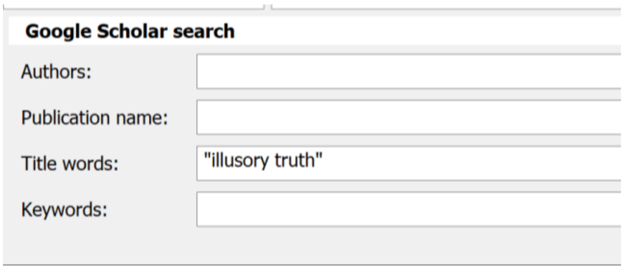

Search 2:

Title words "truth effect"

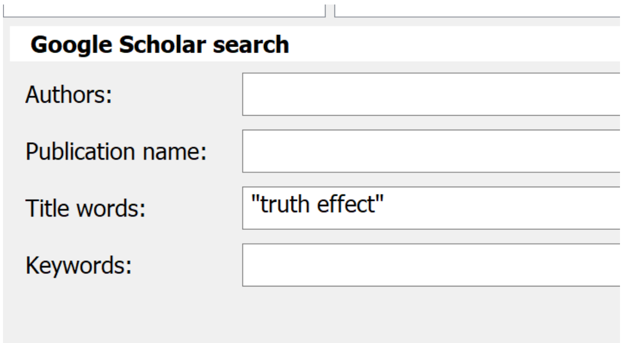

Search 3:

Title words "truth judgement"

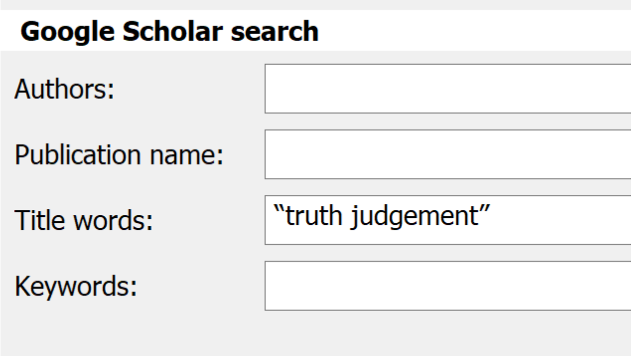

Search 4:

Title words "truth judgment"

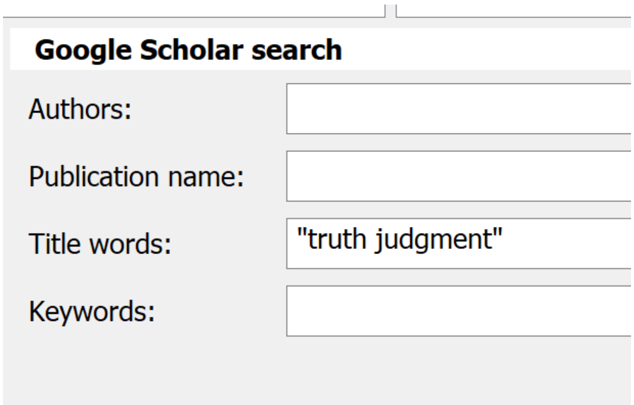

Search 5:
Keywords “illusory truth“

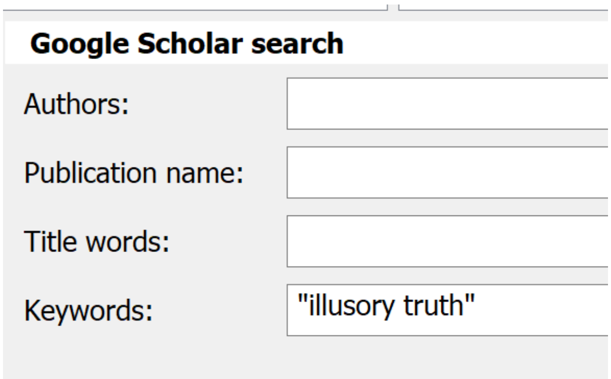

Grey Literature Databases: OpenGrey, PsyArXiv, Curate Science, PsychFileDrawer, DART-Europe, EthOS, ProQuest Dissertation & Theses Global, Thesis Commons
Search 1: “illusory truth“
Search 2: “truth effect”
Search 3: “truth judgement”
Search 4: “truth judgment”

Appendix C

References Included in Full-text Database

	Author	Year	Title
01	Arkes, H.R., Hackett, C., Boehm, L.	1989a	The generality of the relation between familiarity and judged validity
02	Arkes, H. R., Nash, J. G., & Joyner, C. A.	1989b	Solving a word puzzle makes subsequent statements containing the word seem more valid
03	Arkes, Hal R; Boehm, Lawrence E; Xu, Gang	1991	Determinants of judged validity
04	Arkes, H. R., Nash, J. G., & Joyner, C. A.	1993	Replication of solving a word puzzle makes subsequent statements containing the word seem more valid
05	Bacon, F.T.	1979	Credibility of repeated statements: Memory for trivia
06	Bechkoff, J. R.	2008	Proprioception and the truth effect: A case in favor of the cartesian model of information processing
07	Begg, I., Armour, V., & Kerr, T.	1985	On believing what we remember
08	Begg, I., & Armour, V.	1991	Repetition and the ring of truth: Biasing comments
09	Begg, I., Anas, A., & Farinacci, S.	1992	Dissociation of processes in belief: Source recollection, statement familiarity, and the illusion of truth
10	Béna, J., Carreras, O., Terrier, P.	2019a	Attention division and the truth effect: A case of moderation by source credibility manipulation
11	Béna, J., Carreras, O., Terrier, P.	2019b	Delay between exposure and truth judgement decreases the truth effect in a one judgement procedure
12	Béna, J., Carreras, O., Terrier, P.	2020	Does delay between exposure and truth judgement decrease the truth effect through a recollection impairment? A Remember/Know study
13	Boehm, L. E.	1994	The validity effect: A search for mediating variables
14	Brashier, N. M., Umanath, S., Cabeza, R., & Marsh, E. J.	2017	Competing cues: Older adults rely on knowledge in the face of fluency
15	Brashier, N. M., Eliseev, E. D., & Marsh, E. J.	2020	An initial accuracy focus prevents illusory truth
16	Brown, A. S.; Nix, L. A.	1996	Turning lies into truths: Referential validation of falsehoods
17	Calio, F.	2019	Untersuchungen zur zeitlichen stabilität und zur vermeidbarkeit der wahrheitsillusion [Investigations into the temporal stability and the avoidability of the illusion of truth]
18	Chang, Y.	2019	Is the plausibility account of the illusion of truth effect plausible?
19	Corneille, O., Mierop, A., & Unkelbach, C.	2020	Repetition increases both the perceived truth and fakeness of information: An ecological account
20	De keersmaecker, J.; Dunning, D.; Pennycook, G.; Rand, D.G.; Sanchez, C.; Unkelbach, C.; Roets, A.	2020	Investigating the robustness of the illusory truth effect across individual differences in cognitive ability, need for cognitive closure, and cognitive style.
21	Dechêne, A., Stahl, C., Hansen, J., & Wänke, M.	2009	Mix me a list: Context moderates the truth effect and the mere-exposure effect
22	DiFonzo, N., Beckstead, J. W., Stupak, N., & Walders, K.	2016	Validity judgments of rumors heard multiple times: The shape of the truth effect

23	Doland, C. A.	1999	Repeating is believing: an investigation of the illusory truth effect
24	Ecker, U., Lewandowsky, S., & Chadwick, M.	2020	Can corrections spread misinformation to new audiences? Testing for the elusive familiarity backfire effect
25	Effron, D. A., & Raj, M.	2020	Misinformation and morality: encountering fake-news headlines makes them seem less unethical to publish and share
26	Fazio, L. K., Brashier, N. M., Payne, B. K., & Marsh, E. J.	2015	Knowledge does not protect against illusory truth
27	Fazio, L., & Sherry, C.	2019a	The effect of repetition on truth judgments across development
28	Fazio, Lisa K; Rand, David G; Pennycook, Gordon	2019b	Repetition increases perceived truth equally for plausible and implausible statements
29	Fazio, L. K.,	2020a	Repetition increases perceived truth even for known falsehoods
30	Fazio, L. K.	2020b	Preventing the illusory truth effect: When repetition does not increase perceived truth
31	Frances, C.; Costa, A.; Baus, C.	2018	On the effects of regional accents on memory and credibility
32	Garcia-Marques, T., Silva, R. R., Reber, R., & Unkelbach, C.	2015	Hearing a statement now and believing the opposite later
33	Garcia-Marques, T., Silva, R. R., & Mello, J.	2016a	Judging the truth-value of a statement in and out of a deep processing context
34	Garcia-Marques, T., Prada, M., & Mackie, D. M.	2016b	Familiarity increases subjective positive affect even in non-affective and non-evaluative contexts
35	Garcia-Marques, T.; Silva, R.R.; Mello, J.	2017	Asking simultaneously about truth and familiarity may disrupt truth effects
36	Garcia-Marques, T., Silva, R. R., Mello, J., & Hansen, J.	2019	Relative to what? Dynamic updating of fluency standards and between-participants illusions of truth
37	Gigerenzer, G.	1984	External validity of laboratory experiments: The frequency-validity relationship
38	Hasher, L., Goldstein, D., & Toppino, T.	1977	Frequency and the conference of referential validity
39	Hawkins, S. A., & Hoch, S. J.	1992	Low-involvement learning: Memory without evaluation
40	Hawkins, S. A., Hoch, S. J., & Meyers-Levy, J.	2001	Low-involvement learning: Repetition and coherence in familiarity and belief
41	Hernández Vera, A. V.	2020	El efecto de la repetición sobre la percepción de veracidad de frases: un estudio sobre la ilusión de verdad.
42	Jackson, D. R.	2018	Ethics in fake news: combatting the illusory truth effect with corrections
43	Jalbert M., Newman E., & Schwarz N.	2016	Trivia claim truth effect
44	Jalbert M., Newman E., & Schwarz N.	under review	Only half of what I'll tell you is true: How experimental procedures lead to an underestimation of the truth effect
45	Kim, C.	2002	The role of individual differences in general skepticism in the illusory truth effect
46	Ladowsky-Brooks, R. L.	2010	The truth effect in relation to neuropsychological functioning in traumatic brain injury
47	Law, S., & Hawkins, S. A.	1997	Advertising repetition and consumer beliefs: The role of source memory
48	Law, S.	1998a	Do we believe what we remember or, do we remember what we believe?
49	Law, S.	1998b	Investigating the truth effect in young and elderly consumers: The role of recognition and source memory

50	Law, S., Hawkins, S. A., & Craik, F. I.	1998c	Repetition-induced belief in the elderly: Rehabilitating age-related memory deficits
51	Lindsey, S.	1994	Aging and the truth effect in validity judgment
52	Mitchell, J. P.	2003	Asymmetries in the processing of true and false information
53	Mitchell, J. P., Dodson, C. S., & Schacter, D. L.	2005	fMRI evidence for the role of recollection in suppressing misattribution errors: The illusory truth effect
54	Mitchell, J. P., Sullivan, A. L., Schacter, D. L., & Budson, A. E.	2006	Misattribution errors in Alzheimer's disease: The illusory truth effect
55	Moritz, S., Köther, U., Woodward, T. S., Veckenstedt, R., Dechêne, A., & Stahl, C.	2012	Repetition is good? An internet trial on the illusory truth effect in schizophrenia and nonclinical participants
56	Murray, S., Stanley, M., McPhetres, J., Pennycook, G., & Seli, P.	2020	"I've said it before and I will say it again": Repeating statements made by Donald Trump increases perceived truthfulness for individuals across the political spectrum
57	Mutter, S. A., Lindsey, S. E., & Pliske, R. M.	1995	Aging and credibility judgment
58	Nadarevic, L.	2007	A failed replication of the truth effect
59	Nadarevic, L. & Rinnewitz, L.	2011	Judgment mode instructions do not moderate the truth effect
60	Nadarevic, L.; Meckler, D.; Schmidt, A.	2012	Are there interindividual differences of the truth effect? An investigation of different personality traits
61	Nadarevic, L.; Erdfelder, E.	2014	Initial judgment task and delay of the final validity-rating task moderate the truth effect
62	Nadarevic, L., & Aßfalg, A.	2017	Unveiling the truth: warnings reduce the repetition-based truth effect
63	Nadarevic, L., Plier, S., Thielmann, I., & Darancó, S.	2018	Foreign language reduces the longevity of the repetition-based truth effect
64	Newman, E. J., Jalbert, M. C., Schwarz, N., & Ly, D. P.	2020	Truthiness, the illusory truth effect, and the role of need for cognition
65	Oğuz Taşbaş, E. H., Unkelbach, C.,	2020b	Repetition effect and decision making
66	Pennycook, G., & Rand, D. G.	2017	The illusory truth effect for fake news is similar regardless of format
67	Pennycook, G., Cannon, T. D., & Rand, D. G.	2018	Prior exposure increases perceived accuracy of fake news
68	Polage, D.C.	2012	Making up history: False memories of fake news stories
69	Reyes de Luna, B.	2018	La formación de nuestras creencias: Efecto ilusorio de la verdad [The Formation of our beliefs: The illusory truth effect]
70	Roggeveen, A. L., & Johar, G. V.	2002	Perceived source variability versus familiarity: Testing competing explanations for the truth effect
71	Schwartz, Marian	1982	Repetition and rated truth value of statements
72	Silva, R. R., Garcia-Marques, T., & Mello, J.	2016	The differential effects of fluency due to repetition and fluency due to color contrast on judgments of truth
73	Silva, R. R., Garcia-Marques, T., & Reber, R.	2017	The informative value of type of repetition: Perceptual and conceptual fluency influences on judgments of truth
74	Sim, R.	2010	Memory mistakes and aging: How susceptibility to false recognition and the illusory truth effect changes across the lifespan
75	Skurnik, I. W.	1998	Metacognition and the illusion of truth
76	Skurnik, I., Yoon, C., Park, D. C., & Schwarz, N.	2005	How warnings about false claims become recommendations

77	Srull, T. K.	1983	The role of prior knowledge in the acquisition, retention, and use of new information
78	Stanley, M. L., Yang, B. W., & Marsh, E. J.	2019	When the unlikely becomes likely: Qualifying language does not influence later truth judgments
79	Sundar, A.; Kardes, F.R.; Wright, S.A.	2015	The influence of repetitive health messages and sensitivity to fluency on the truth effect in advertising
80	Toppino, T. C., Robertshaw, W., Hasher, L., & Goldstein, D.	1977	Frequency of occurrence and judgments of truth and falsity
81	Toppino, T.C.; Ann Brochin, H.	1989	Learning from tests: The case of true-false examinations
82	Toppino, T.C.; Luipersbeck, S.M.	1993	Generality of the negative suggestion effect in objective tests
83	Ulenaers, W., Pieters, R., & Warlop, L.	2000	Felt expertise and the truth effect
84	Unkelbach, C.	2007	Reversing the truth effect: Learning the interpretation of processing fluency in judgments of truth
85	Unkelbach, C., & Stahl, C.	2009	A multinomial modeling approach to dissociate different components of the truth effect
86	Unkelbach, C., Bayer, M., Alves, H., Koch, A., & Stahl, C.	2011	Fluency and positivity as possible causes of the truth effect
87	Unkelbach, C., & Rom, S. C.	2017	A referential theory of the repetition-induced truth effect
88	Unkelbach, C.; Greifeneder, R.	2018	Experiential fluency and declarative advice jointly inform judgments of truth
89	Unkelbach, C., & Speckmann, F.	2020a	Acting on illusory truth despite knowing better
90	Unkelbach	2020b	Knowledge does partially protect against illusory truth: The case of information related to the Corona crisis
91	Vicari, S. M.	2016	Overcoming the illusory truth effect: The influence of contextual details on memory monitoring
92	Vogel, Tobias; Silva, Rita R; Thomas, Aurelia; Wanke, Michaela	2020	Truth is in the mind, but beauty is in the eye: Fluency effects are moderated by a match between fluency source and judgment dimension
93	Wang, W. C., Brashier, N. M., Wing, E. A., Marsh, E. J., & Cabeza, R.	2016	On known unknowns: Fluency and the neural mechanisms of illusory truth

Appendix D

Summary of Statcheck Issues

N errors	N papers	% papers	N decision errors	N papers	% papers
0	31	33.3	0	53	57
1	12	12.9	1	2	2.2
2	8	8.66	3	2	2.2
3	2	2.2			
4	1	1.1			
5	2	2.2			
9	1	1.1			
Unable to read	36	38.7			

Note. Statcheck recomputes p-values and compares them to those reported in the text. Inconsistent p-values are recorded as an “error”. If the reported result is significant and the recomputed result is not, or vice versa, the result is recorded as a “decision error”.
